# Genomic Prediction of Yield Traits in Single-Cross Hybrid Rice (*Oryza sativa* L.)

**DOI:** 10.3389/fgene.2021.692870

**Published:** 2021-06-30

**Authors:** Marlee R. Labroo, Jauhar Ali, M. Umair Aslam, Erik Jon de Asis, Madonna A. dela Paz, M. Anna Sevilla, Alexander E. Lipka, Anthony J. Studer, Jessica E. Rutkoski

**Affiliations:** ^1^Department of Crop Sciences, University of Illinois at Urbana-Champaign, Urbana, IL, United States; ^2^Rice Breeding Platform, International Rice Research Institute, Los Baños, Philippines

**Keywords:** hybrid rice, genomic selection, general combining ability, breeding, best linear unbiased prediction

## Abstract

Hybrid rice varieties can outyield the best inbred varieties by 15 – 30% with appropriate management. However, hybrid rice requires more inputs and management than inbred rice to realize a yield advantage in high-yielding environments. The development of stress-tolerant hybrid rice with lowered input requirements could increase hybrid rice yield relative to production costs. We used genomic prediction to evaluate the combining abilities of 564 stress-tolerant lines used to develop Green Super Rice with 13 male sterile lines of the International Rice Research Institute for yield-related traits. We also evaluated the performance of their F_1_ hybrids. We identified male sterile lines with good combining ability as well as F_1_ hybrids with potential further use in product development. For yield per plant, accuracies of genomic predictions of hybrid genetic values ranged from 0.490 to 0.822 in cross-validation if neither parent or up to both parents were included in the training set, and both general and specific combining abilities were modeled. The accuracy of phenotypic selection for hybrid yield per plant was 0.682. The accuracy of genomic predictions of male GCA for yield per plant was 0.241, while the accuracy of phenotypic selection was 0.562. At the observed accuracies, genomic prediction of hybrid genetic value could allow improved identification of high-performing single crosses. In a reciprocal recurrent genomic selection program with an accelerated breeding cycle, observed male GCA genomic prediction accuracies would lead to similar rates of genetic gain as phenotypic selection. It is likely that prediction accuracies of male GCA could be improved further by targeted expansion of the training set. Additionally, we tested the correlation of parental genetic distance with mid-parent heterosis in the phenotyped hybrids. We found the average mid-parent heterosis for yield per plant to be consistent with existing literature values at 32.0%. In the overall population of study, parental genetic distance was significantly negatively correlated with mid-parent heterosis for yield per plant (*r* = −0.131) and potential yield (*r* = −0.092), but within female families the correlations were non-significant and near zero. As such, positive parental genetic distance was not reliably associated with positive mid-parent heterosis.

## Introduction

Hybrid crop varieties are economically valued for increased vigor, yield, yield stability, and uniformity in species including maize, sugar beet, and cotton (Hochholdinger and Baldauf, 2018). Rice (*Oryza sativa L.*) is a self-pollinated crop that has traditionally been cultivated as an inbred, but the introduction of male sterility into cultivated germplasm in the 1970s enabled hybrid breeding (Virmani and Wan, 1988; Yuan et al., 1989; Nalley et al., 2017). Public hybrid rice varietal development to date has resulted primarily from identification of superior single crosses rather than the systematic breeding of heterotic pools (Lu and Xu, 2010; Spielman et al., 2013). Developing heterotic pools for rice by reciprocal recurrent selection methods may increase the rate of genetic gain for hybrid rice breeding compared to evaluating random crosses, because reciprocal recurrent selection can concurrently improve the additive value of the populations while exploiting heterosis due to dominance (Comstock et al., 1949).

Existing hybrid rice varieties may outyield the best inbred varieties by 10 to 30% with appropriate management (Spielman et al., 2013). However, adoption of hybrid rice varieties is low outside of China, in part because the hybrid yield advantage of temperate *japonica* varieties used in China is much greater than that observed in tropical *indica* varieties to date (Janaiah and Xie, 2010; Longin et al., 2012; Spielman et al., 2013). In some countries, socioeconomic factors such as lack of irrigation systems, paved roads, certified seed suppliers, seed marketing, farmer education, and available credit to purchase seed and fertilizer have limited hybrid rice adoption (Mottaleb et al., 2015; Abebrese et al., 2019). Of the agronomic factors that influence hybrid rice adoption, poor grain quality has been a longstanding challenge, but breeding progress since the early 2000s has produced some acceptable varieties (Spielman et al., 2013). Surveys of farmers suggest that poor quality is not the primary determinant of hybrid rice rejection (Spielman et al., 2013; Feng et al., 2017). Farmers choose not to grow hybrid rice for many reasons, including the high cost of seed, poor seed quality, and lack of hybrid seed availability (Spielman et al., 2013). However, the key agronomic reason for limited adoption is that hybrid rice varieties require more intensive management of irrigation, fertilizer, weeds, and other biotic stressors to provide a yield advantage over inbred varieties in otherwise high-yielding environments (Spielman et al., 2013; Mottaleb et al., 2015; Nalley et al., 2016). Therefore, the development of stress-tolerant hybrids with lowered input requirements could spur hybrid adoption and unlock hybrid yield advantages.

In this study, we evaluated the general combining abilities (GCAs) of the existing male sterile lines of the International Rice Research Institute (IRRI) with stress-tolerant germplasm used in the development of Green Super Rice varieties, as well as the performance, or genetic value, of their F_1_ hybrids (Sprague and Tatum, 1942; Ali et al., 2018; Yu et al., 2020). The founders of the Green Super Rice program were selected for multiple stress tolerances, including salinity, submergence, tungro disease, anaerobic germination conditions, and low water and nitrogen inputs (Ali et al., 2018). We sought to identify any outstanding F_1_ hybrids—which may be as stress-tolerant and yet higher-yielding than existing Green Super Rice lines—to advance for further testing for varietal release. We also sought to identify male and female lines with superior GCA which could be used to initiate the development of heterotic pools from IRRI germplasm, presumably stacked with alleles conferring stress tolerance. In addition to phenotypic evaluation, we used genomic prediction to evaluate non-phenotyped parental lines and hybrid crosses.

We also tested whether parental genetic distance was correlated with mid-parent heterosis using a large sample of hybrids and genome-wide molecular markers. Mid-parent heterosis due to dominance is expected to be positively correlated with parental squared difference in allele frequency (SDAF) by quantitative genetic theory (Falconer and Mackay, 1996; Amuzu-Aweh et al., 2013). It has also been posited that genetic divergence in founders of heterotic pools may lead to improved gain in reciprocal recurrent selection programs, even though in practice heterotic pools have been developed from closely related germplasm in species such as maize (Melchinger, 1999; Tracy and Chandler, 2006; Rembe et al., 2019). A previous study of rice which used > 100k genome wide markers and six parental lines found a curvilinear relationship of genetic distance and mid-parent heterosis, with mid-parent heterosis increasing with genetic distance to a point and then declining (Waters et al., 2015). Due to past lack of availability of molecular markers, other studies used fewer than 500 markers and found positive correlations of genetic distance and heterosis using 10 or 22 parents (Xiao et al., 1996; Kwon et al., 2002). However, another study using 319 markers found no correlation of genetic distance and heterosis in progeny of 13 parents (Boeven et al., 2020). In other species, such as wheat, whether parental genetic distance is correlated with heterosis varies, with different findings among studies and populations (Melchinger, 1999; Boeven et al., 2020). We wished to test whether parents of hybrids with high SDAF tended to produce hybrids with high mid-parent heterosis in our rice population of study.

## Materials and Methods

### Plant Materials and Population Design

The plant materials for prediction comprised 13 female lines, 564 male lines, and their 10,716 possible F_1_ hybrids (Supplementary File 1). Twelve of the female lines were wild-abortive cytoplasmic male sterile (CMS), and one female line, A07, was thermosensitive genic male sterile (TGMS). The 564 male tester lines were backcross introgression lines (BILs) from 11 families. Each family of BILs was generated by crossing one of the 11 diverse males to a common female, Weed Tolerant Rice 1 (WTR-1), and advancing the backcrosses to the BC_1_F_5_ generation under stringent selection for multiple stress tolerances as described in Ali et al. (2018). The recurrent parent, WTR-1, was a restorer line, but the male lines likely segregated for fertility restoration.

Of the 10,716 possible F_1_ hybrids, a random subset of 938 were made to comprise the genomic prediction model training set by crossing six female lines to 137 males. To avoid unintentional selection for synchronous flowering, the female parents had two planting dates. None of the 137 male lines were completely crossed to all six females. However, in pairwise comparisons of females, all females had some overlap with each other female in males crossed (Supplementary Table 1). In total, 85 of the males were crossed to a single female, 108 of the males were crossed to 2 females, 124 of the males were crossed to 3 females, 60 of the males were crossed to 4 females, and 5 of the males were crossed to 5 females. All female lines were manually emasculated to prevent contamination by selfing and to expose the stigma.

Two groups of lines were used to estimate mid-parent heterosis and commercial relative performance but were not included for prediction. The five maintainer (B) lines of the five CMS female parents were used to estimate mid-parent heterosis. Six inbred lines were used as commercial checks to estimate commercial relative performance: five of the donor parents, Y 134 (DP 6), Khazar (DP 8), OM 997 (DP 10), M 401 (DP17), and X 21 (DP19), and the recurrent parent, WTR-1.

### Field Experimental Design

The F_1_ hybrids, their inbred parents, the female maintainer lines, and the commercial checks were phenotyped in an unbalanced randomized complete block design (RCBD) in two environments, irrigated lowland and irrigated upland, at the IRRI farm (approximately 14°09′50.7″ N, 121°15′50.5″ E) in the dry season of 2018. After establishment in seedbeds on January 8, 2018, seedlings were transplanted at the three-leaf stage. Transplanting occurred on January 31, 2018, at the irrigated lowland site and on February 8, 2018, at the irrigated upland site. The plants were harvested the week of May 14, 2018. Basal NPK fertilizer was applied at a rate of 30 kg/ha, and zinc was applied at a rate of 5 kg/ha. N fertilizer was also applied at 28–30 days after transplanting and at the panicle initiation stage (42 days after transplanting) at a rate of 35 kg/ha. Rat fences were installed at both locations; bird pressure was controlled by farmworkers in the lowland environment, and by a bird net at the upland environment. Both environments were hand-weeded. Both environments were irrigated, but the lowland environment was continuously flooded to a depth of ∼10 cm, whereas water depth was allowed to vary in the upland field. Insect pressure was controlled by application of Regent^®^ pesticide (fipronil). Temperatures were sufficient throughout the growing season to ensure seed set in the TGMS female line A07.

The field layout was designed in PBTools 1.4, which depends on the R package agricolae (IRRI, 2014; de Mendiburu, 2020). There were two blocks per environment with one replicate per genotype per block, but replicates were missing for some genotypes. Genotypes were replicated in single-row plots due to limited availability of hybrid seed, with five plants per row, and plants were spaced to 25 × 20 cm within rows. Measurements were only recorded for plants at 20 cm spacing within rows; i.e., edge plants were not measured, nor were plants with missing neighbor plants within the row.

The following traits or trait derivatives were phenotyped: plant height, number of tillers, panicle dry weight, panicle length, proportion of spikelets filled, yield per plant, and yield potential per plant (Supplementary File 1). For all genotype replicates, phenotypes were averaged across measured plants in a single-row plot; plants were subsamples, but were not treated as subsamples in downstream modeling because subsampling variance was not of interest. Plant height was measured from the base of the plant to the panicle tip after flowering. All tillers were assumed to be productive based on observations in a subset of samples. For panicle measurements and yield estimates, three random panicles were harvested from each sampled plant, totaling nine panicles per replicate. Panicle length was measured from the pedicel to the panicle tip and averaged across all panicles in a replicate. For a given replicate, yield per plant was calculated as panicle dry weight times tiller number. For a given replicate, yield potential per plant was calculated as average panicle dry weight divided by proportion of spikelets filled times tiller number. Yield potential per plant and proportion of spikelets filled were only measured in the irrigated lowland environment due to cost.

### Molecular Marker Generation and Analysis

Genome-wide molecular markers were generated for the parents with tunable genotyping-by-sequencing^®^ (tGBS) by data2Bio and its subsidiary, Freedom Markers, in Ames, Iowa (Ali et al., 2017; Ott et al., 2017). In general, each individual DNA sample was double-digested with restriction enzymes, then the resulting fragments were ligated to a uniquely barcoded adapter at the 5′ end. At the 3′ end, the fragments were ligated to a universal sequencing adapter. However, in the first subsequent PCR amplification of the library, the complementary primer for the universal sequencing adapter was extended by 1-3 nucleotides; only fragments in which the genomic sequence complemented the extension were amplified. Then, the libraries were amplified with Ion Proton sequencing primers.

The male and female parents were sequenced in separate Ion Proton runs. For the male parents, a total of ten Ion Proton runs were done; the female parents were sequenced in two Ion proton runs as part of a larger set. The Ion Proton sequencing reads were trimmed by the manufacturer to remove adapter sequence and bases with PHRED quality scores less than 15 in the software Lucy (Chou and Holmes, 2001). For the female parent A07, additional RAD-sequencing was done in-house. In brief, DNA was extracted from mature leaf tissue of the A07 parent and digested separately with one of three enzyme combinations: *Ape*KI-*Pst*I, *Hin*P1I-*Pst*I, or *Ape*KI only. Then, each digestion was ligated separately to unique barcoded adapters and subsequently pooled. Fragments were selected for sizes ranging from 200–500 bp, and the libraries were amplified by PCR. Then, the libraries were sequenced for single-read 100 bp reads with an Illumina NovaSeq6000 SP. All reads were aligned to the Nipponbare IRGSP-1.0 v7 reference genome in GSNAP 2017-11-15 (Kawahara et al., 2013; Wu et al., 2016). Then, variants were called in BCFtools 1.7 (SAMtools, 2018).

**FIGURE 1:**
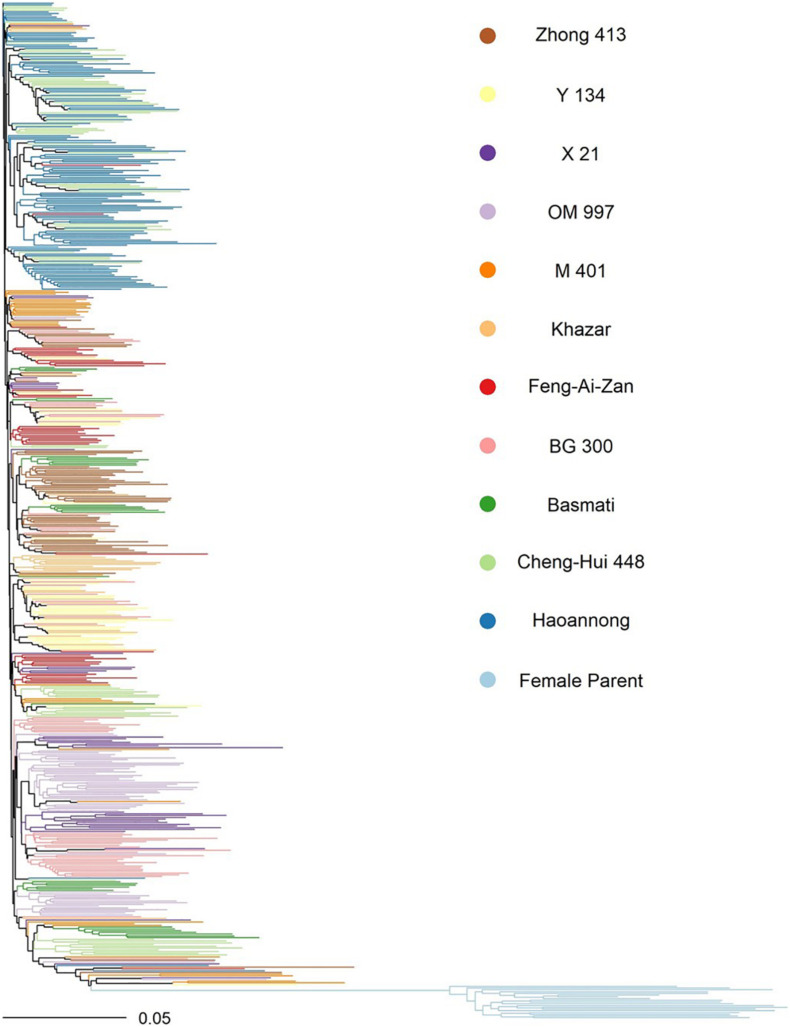
Neighbor-joining tree of the parental lines used in the study. Color indicates the female parents (light blue) and, for the male parents, their subfamily by donor parent (all other colors). The recurrent parent for all males was WTR-1.

Variant sites were filtered separately within the male and female parent sets in TASSEL 5.0 (Glaubitz et al., 2014). Within sets, only the 2 most major alleles of the variant were considered, and at most 50% of the individuals were permitted to be heterozygous. In the females, the minimum site count was 2 (corresponding to a minimum site presence of 10% in all females), and the minimum minor allele count was 2, yielding 77,709 polymorphic sites among the females. In the males, the minimum site count was 188 (corresponding to a minimum site presence of 33% in the males), and the minimum minor allele count was 3, yielding 148,922 total polymorphic sites among the males. The genotypes were imputed separately for males and females in Beagle 5.0 (Browning et al., 2018). Principal components analysis of the male and female parent genotypes was done using 20,000 sites common to both using the *glPca* function in the R package adegenet (Jombart and Ahmed, 2011). The F_1_ hybrid genotypes were inferred from the same 20,000 sites common to males and females using the *build.HMM* function in the R package sommer version 3.8 (Covarrubias-Pazaran, 2016, 2018). A phylogenetic neighbor-joining tree of the male and female parent genotypes was constructed in TASSEL 5.0 using the same common 20,000 sites, and the tree was visualized in the R package ape version 5.5 (Figure 1; Paradis and Schliep, 2019).

The additive relationship matrices of each the females, males, and F_1_ hybrids, denoted respectively as **G_F_**, **G_M_**, and **G_H_**, were calculated with the *A.mat* function in sommer by the method of Endelman and Jannink (2012). The 77,709 imputed female sites were used to estimate **G_F_**, and the 148,922 imputed male sites were used to estimate **G_M_**. The hybrid genotypes inferred from the 20,000 sites common to males and females via the *build.HMM* function were used to estimate **G_H_**. The specific combining ability (SCA) relationship matrix **G_FM_** was the Kronecker product of the male and female additive relationship matrices (Bernardo, 2002). Pairwise SDAF, a hypothetical predictor of mid-parent heterosis, was calculated as:

(1) $$S\,D\,A\,F_{i\,j}=\frac{\sum_{n=1}^{N}{\left(p_{i_{n}}-p_{j_{n}}\right)}^{2}}{N}\;$$

Where *SDAF*_*ij*_ was the squared difference in allele frequency between the *i*^th^ female parent and the *j*^th^ male parent, *p_i_n__*-*p_j_n__* was the difference allele frequency between the *i*^th^ female parent and *j*^th^ male parent at the *n*^th^ variant site, and *N* was the total number of variant sites.

### Modeling and Statistical Analysis

All linear models were fit in a single step with the *mmer* function in sommer (Covarrubias-Pazaran, 2016, 2018). For a given trait, i.e., plant height, tiller number, panicle length, average yield per plant, proportion of spikelets filled, or potential yield per plant, the genotype replicates (i.e., single-row plots) were considered the experimental unit. Genetic variances for plant height, tiller number, panicle length, and average yield per plant traits were estimated with models of the following form:

(2) $$Y_{i\,j\,k\,l}=\mu +H_{\text{i}}+E_{j}+H\,E_{\text{ij}}+B_{\left(k\right)\,j}+\varepsilon_{i\,j\,k\,l}\;$$

where *Y*_*ijkl*_ was the random phenotypic response of the *i*^th^ single-cross hybrid genotype in the *k*^th^ block nested in the *j*^th^ environment from the *l*^th^ replicate, μ was the grand mean, *H*_i_ was the random effect of the *i*^th^ hybrid genotype with $N\,\left(0,\text{I}\,\sigma_{H}^{2}\right)$, *E*_*j*_ was the random effect of the *j*^th^ environment with $N\,\left(0,\text{I}\,\sigma_{E}^{2}\right)$, *HE*_ij_ was the random interaction of the *i*^th^ hybrid genotype and the *j*^th^ environment with $N\,\left(0,\text{I}\,\sigma_{H\,E}^{2}\right)$, *B*_*(k)j*_ was the effect of the *k*^th^ block nested within the *j*^th^ environment with $N\,\left(0,\text{I}\,\sigma_{B}^{2}\right)$, and ε_*ijkl*_ was the random error associated with each replicate with N(0,Iσ)e2.

The genetic variance for proportion of spikelets filled and potential yield per plant was estimated using the following model in (3), without the environment term and its associated interactions, because the traits were only phenotyped in the irrigated lowland environment:

(3) $$Y_{i\,j\,k}=\mu +H_{\text{i}}+B_{j}+\varepsilon_{i\,j\,k}\;$$

*Y*_*ijk*_ was the random phenotypic response of the *i*^th^ hybrid genotype in the *j*^th^ block from the *k*^th^ replicate,μ was the grand mean, *H*_i_ was the random effect of the *i*^th^ hybrid genotype with $N\,\left(0,\text{I}\,\sigma_{H}^{2}\right)$, *B*_*j*_ was the random effect of the *j*^th^ block with $N\,\left(0,\text{I}\,\sigma_{B}^{2}\right)$, and ε_*ijk*_ was the random error associated with each replicate with N(0,Iσ)e2.

The entry-mean heritability was estimated for each of height, tiller number, panicle length, and yield per plant by (4) following the method of Holland et al. (2003) for unbalanced RCBDs:

(4) $$H^{2}=\frac{\sigma_{H}^{2}}{\sigma_{H}^{2}+\frac{\sigma_{H\,E}^{2}}{h_{j}}+\frac{\sigma_{e}^{2}}{h_{t}}}\;$$

where $\sigma_{H}^{2}$ was the variance among hybrid genotypes from models of the form in (2), $\sigma_{H\,E}^{2}$ was the variance of the interaction of the hybrid genotype and environment, $\sigma_{e}^{2}$ was the error variance, *h*_*j*_ was the harmonic mean of the number of total observations of each hybrid genotype within an environment, and *h*_*t*_ was the harmonic mean of the total number of observations per hybrid genotype.

For proportion of spikelets filled and potential yield, which were phenotyped in a single environment, entry-mean heritability was estimated by (5) also following Holland et al. (2003), using the following equation with the terms as described in (3):

(5) $$H^{2}=\frac{\sigma_{H}^{2}}{\sigma_{H}^{2}+\frac{\sigma_{e}^{2}}{h_{t}}}\;$$

Additive genetic variances were estimated from models of the form in (6) for plant height, tiller number, panicle length, and yield per plant. The terms of (6) are the same as in (2), but in (6) the random effect *H* was assumed to have a multivariate normal (MVN) distribution with $H∼M\,V\,N\,\left(0,{\text{G}}_{\text{H}}\,\sigma_{H}^{2}\right)$, where **G**_H_ was the additive genomic relationship matrix of the F_1_ hybrids:

(6) $$Y_{i\,j\,k\,l}=\mu +H_{\text{i}}+E_{j}+H\,E_{\text{ij}}+B_{\left(k\right)\,j}+\varepsilon_{i\,j\,k\,l}\;$$

Additive genetic variances for proportion of spikelets filled and potential yield were estimated from model of the form in (7), with the same terms as (3), but the random effect *H* was assumed to have a multivariate normal (MVN) distribution with $H∼M\,V\,N\,\left(0,{\text{G}}_{\text{H}}\,\sigma_{H}^{2}\right)$, where **G_H_** was the additive genomic relationship matrix of the F_1_ hybrids:

(7) $$Y_{i\,j\,k}=\mu +H_{\text{i}}+B_{j}+\varepsilon_{i\,j\,k}\;$$

Narrow-sense heritability, or the proportion of additive genetic variance out of total phenotypic variance, was estimated on a single-plant basis for all traits. Variance components were estimated from the models of the form in (6) for plant height, tiller number, panicle length, and yield per plant, and narrow-sense heritability was estimated with (8):

(8) $$h^{2}=\frac{\sigma_{H}^{2}}{\sigma_{H}^{2}+\sigma_{H\,E}^{2}+\sigma_{e}^{2}}\;$$

For proportion of spikelets filled and potential yield, narrow-sense heritability was estimated using (9) with variances estimated from the models of the form in (7):

(9) $$h^{2}=\frac{\sigma_{H}^{2}}{\sigma_{H}^{2}+\sigma_{e}^{2}}\;$$

For each trait, genomic best linear unbiased predictions (GBLUPs) of hybrid genetic value, male GCA, and female GCA were each estimated using two separate models: the genomic GCA model and the genomic GCA + SCA model. Model fits were compared with the Akaike information criterion (AIC) and Bayesian information criterion (BIC). The genomic GCA model was:

(10) $$Y_{i\,j\,k\,l\,m}=\mu +F_{i}+M_{j}+E_{k}+B_{\left(l\right)\,k}+F\,E_{i\,k}+M\,E_{j\,k}+\varepsilon_{i\,j\,k\,l\,m}\;$$

where *Y*_*ijklm*_ was the random phenotypic response of a single-cross hybrid of the *i*^th^ female line and the *j*^th^ male line observed in the *k*^th^ environment, *l*^th^ block, and *m*^th^ replicate, μ was the grand mean, *F*_*i*_ was the random GCA effect of the *i*^th^ female parent with $F∼M\,V\,N\,\left(0,{\text{G}}_{\text{F}}\,\sigma_{F}^{2}\right)$ where **G**_F_ was the additive genomic relationship matrix among females, *M*_*j*_ was the random GCA effect of the *j*^th^ male parent with $M∼M\,V\,N\,\left(0,{\text{G}}_{\text{M}}\,\sigma_{M}^{2}\right)$ where **G**_M_ was the additive genomic relationship matrix among males, *E*_*k*_ was the effect of the *k*^th^ environment with $N\,\left(0,\text{I}\,\sigma_{E}^{2}\right)$, *B*_*(t)k*_ was the effect of the *l*^th^ block nested in the *k*^th^ environment with $N\,\left(0,\text{I}\,\sigma_{B}^{2}\right)$, *FE*_*ik*_ was the random interaction of the *i*^th^ female and the *k*^th^ environment with $N\,\left(0,\text{I}\,\sigma_{F\,E}^{2}\right)$, *ME*_*jk*_ was the random interaction of the *j*^th^ male and the *k*^th^ environment with $N\,\left(0,\text{I}\,\sigma_{M\,E}^{2}\right)$, and ε_*ijklm*_ was the random error of each observation with N(0,Iσ)e2.

The genomic GCA + SCA model was:

(11) $$Y_{i\,j\,k\,l\,m}=\mu +F_{i}+M_{j}+E_{k}+B_{\left(l\right)\,k}+F\,E_{i\,k}+M\,E_{j\,k}+F\,M_{i\,j}+\varepsilon_{i\,j\,k\,l\,m}$$

where terms were as described above, and *FM*_*ij*_ was the additional random SCA interaction effect of the *i*^th^ female and the *j*^th^ male, with $F\,M∼M\,V\,N\,\left(0,{\text{G}}_{\text{FM}}\,\sigma_{F\,M}^{2}\right)$. **G**_FM_ was the Kronecker product of **G**_F_ and **G**_M_ (Bernardo, 2002).

Best linear unbiased predictions (BLUPs) of hybrid genetic value and male and female GCAs were also estimated without genomic information to 1) estimate the predictive ability and prediction accuracy of the genomic prediction models, and 2) estimate the accuracy of phenotypic selection. For the GCA model, all terms remained the same as in (10), but the distribution of the random effects of *F* and *M* were simply assumed to be $N\,\left(0,\text{I}\,\sigma_{F}^{2}\right)$ and $N\,\left(0,\text{I}\,\sigma_{M}^{2}\right)$ respectively.

(12) $$Y_{i\,j\,k\,l\,m}=\mu +F_{i}+M_{j}+E_{k}+B_{\left(l\right)\,k}+F\,E_{i\,k}+M\,E_{j\,k}+\varepsilon_{i\,j\,k\,l\,m}$$

Similarly, for the GCA + SCA model, all terms remained the same as in (11), but the distribution of the random effects *F*, *M* and *FM* were assumed to be $N\,\left(0,\text{I}\,\sigma_{F}^{2}\right)$, $N\,\left(0,\text{I}\,\sigma_{M}^{2}\right)$, and $N\,\left(0,\text{I}\,\sigma_{F\,M}^{2}\right)$ respectively:

(13) $$Y_{i\,j\,k\,l\,m}=\mu +F_{i}+M_{j}+E_{k}+B_{\left(l\right)\,k}+F\,E_{i\,k}+M\,E_{j\,k}+F\,M_{i\,j}+\varepsilon_{i\,j\,k\,l\,m}$$

There are multiple methods to estimate genomic prediction accuracy (Estaghvirou et al., 2013). Here, predictive ability was Pearson’s correlation of an estimated value and a true value. Prediction accuracy was considered to be predictive ability divided by the square root of the reliability of the estimated value (Mrode, 2014). This method of estimating prediction accuracy, which is well-established in animal breeding programs, was chosen because it is relatively unbiased, precise, and stable compared to other methods (Estaghvirou et al., 2013). Predictive abilities of the genomic GCA and genomic GCA + SCA models described in (10) and (11) were each estimated for male GCA by ten-fold cross-validation (Resende et al., 2012). Sample size was inadequate to estimate genomic predictive ability and accuracy for female GCA. In each fold, the hybrid progeny phenotypes of 38 of the males were masked from the training set, and the masked training set was used to train the prediction model. Predictive abilities for male GCA, or Pearson’s correlation of the GBLUP of male GCA estimated in the training set fold and the BLUP of male GCA estimated from all available observations in the full dataset, were then calculated for the 38 masked males and averaged across folds for each model. Prediction accuracy for male GCA for each model was the predictive ability divided by the square root of the reliability of the genomic prediction. For each model, the square root of the reliability of the genomic prediction was the correlation of the GBLUP of male GCA and BLUP of male GCA when all available observations were used for estimation of both, as in (10), (11), (12), and (13).

Predictive abilities and prediction accuracies were also estimated for hybrid genetic values for each phenotypic response following Technow et al. (2014). For each of 500 replications, four females and 127 males which had been crossed to at least one of the four females were randomly sampled. Then, a training set was formed by randomly sampling 150 hybrids which had phenotypic records available, with the constraint that the randomly sampled male and female lines had at least one hybrid progeny in the training set. Predictive ability, here Pearson’s correlation of the hybrid genetic values estimated from the training fold and the observed hybrid genetic values, was recorded for hybrids which were not included in the training set. Predictive ability was recorded separately for hybrids which had both parents included in the training set (T2), one parent included in the training set (T1), only the female parent included in the training set (T1F), only the male parent included in the training set (T1M), and neither parent included in the training set (T0). The prediction accuracy was the predictive ability divided by the square root of the reliability of the genomic prediction, here the correlation of the genomic BLUP of hybrid genetic value and BLUP of hybrid genetic value when all available observations were used for estimation of both.

The accuracies of phenotypic selection for each trait were estimated for male GCA, female GCA, and hybrid genetic value as the square root of the reliabilities of their respective BLUPs (Falconer and Mackay, 1996; Mrode, 2014). Reliabilities of male GCA and female GCA were estimated from each the GCA model in (12) and the GCA + SCA model in (13). Reliabilities of hybrid genetic values were estimated from model (2) and (3). To estimate each reliability, the prediction error variances (*PEV*) of the appropriate BLUPs (i.e., of hybrid genetic value, female GCA, or male GCA) were obtained in sommer by inverting the coefficient matrix of the relevant model. For the BLUPs of male GCA for a given model and trait, the reliability was the average of:

(14) $$1-\frac{P\,E\,V_{j}}{\sigma_{M}^{2}}\;$$

where *PEV*_*j*_ was the prediction error variance of the *j*^th^ BLUP of male GCA and $\sigma_{M}^{2}$ was the estimated male GCA variance. For the BLUPs of female GCA for a given model and trait, the reliability was the average of:

(15) $$1-\frac{P\,E\,V_{i}}{\sigma_{F}^{2}}\;$$

where *PEV*_*i*_ was the prediction error variance of the *i*^th^ BLUP of female GCA, and $\sigma_{F}^{2}$ was the estimated female GCA variance. For the BLUPs of hybrid genetic value for a given trait from model (2), the reliability was the average of:

(16) $$1-\frac{P\,E\,V_{i}}{\sigma_{H}^{2}}\;$$

where *PEV*_*i*_ was the prediction error variance of the *i*^th^ BLUP of hybrid genetic value, and $\sigma_{H}^{2}$ was the estimated variance among hybrids.

To assess correlation of SDAF with mid-parent heterosis, mid-parent heterosis of each F_1_ hybrid was estimated using BLUPs of genetic values from the following model:

(17) $$Y_{i\,j\,k\,l\,m}=\mu +F_{i}+G_{j}+E_{k}+G\,E_{j\,k}+B_{\left(l\right)\,k}+\varepsilon_{i\,j\,k\,l\,m}\;$$

*Y*_*ijklm*_ was the random phenotypic response and μ was the grand mean. In the vein of Xiang et al. (2016) and Liang et al. (2018), *F*_*i*_ was the fixed effect of an indicator of whether the genotype was an inbred or F_1_ hybrid to account for the possibility of differing inbred and hybrid group means in the presence of heterosis. *G*_*j*_ was the random effect of the *j*^th^ inbred parent, inbred commercial check, or F_1_ hybrid genotype with $N\,\left(0,\text{I}\,\sigma_{G}^{2}\right)$ *E*_*k*_ was the random effect of the *k*^th^ environment with $N\,\left(0,\text{I}\,\sigma_{E}^{2}\right)$, *GE*_*jk*_ was the random interaction effect of the *j*^th^ genotype and the *k*^th^ environment with $N\,\left(0,\text{I}\,\sigma_{G\,E}^{2}\right)$, *B*_*(l)*_ was the random effect of the *l*^th^ block nested within the *k*^th^ environment with $N\,\left(0,\text{I}\,\sigma_{B}^{2}\right)$, and ε_*ijklm*_was the random error associated with each observation with $N\,\left(0,\text{I}\,\sigma_{e}^{2}\right)$. The environment term and its associated interactions were dropped in estimation of yield potential and proportion of spikelets filled, because they were observed only in the irrigated lowland environment.

Mid-parent heterosis of each F_1_ hybrid was obtained as:

(18) $$M\,P\,H=\frac{\hat{H}-\hat{M\,P}}{\hat{M\,P}}\;$$

where *MPH* was mid-heterosis, $\hat{H}$ was the BLUP of the F_1_ genotype value from (17), and $\hat{M\,P}$ was the mid-parent value, i.e., the mean of the BLUPs from (17) of its parental genotype values (Supplementary File 1). The BLUP of the *i*^th^ genotype value was the sum of μ, ${\hat{F}}_{i}$, and${\hat{G}}_{\text{j}}$ from (17). Because the CMS parents do not set seed, the corresponding maintainer (B) line phenotype was used to estimate heterosis. Pearson’s correlation of SDAF with mid-parent heterosis was estimated among all hybrids in the study and also separately within families of hybrids with the same female parent. Student’s *t* test of significance was conducted at α = 0.05 for each correlation, with the null hypothesis that a given correlation did not significantly differ from zero and the alternate hypothesis that the given correlation significantly differed from zero.

Commercial relative performance (commercial heterosis) was estimated for each F_1_ hybrid with phenotypic observations against each check as:

(19) $$C\,R\,P=\frac{\hat{H}-\hat{C}}{\hat{C}}\;$$

where *CRP* was commercial relative performance, $\hat{H}$ was the BLUP of the F_1_ hybrid genotype value from (17), and $\hat{C}$ was the BLUP of the commercial check genotype value from (17). The BLUP of the *i*^th^ genotype value was the sum of μ, ${\hat{F}}_{i}$, and${\hat{G}}_{\text{j}}$ from (17).

## Results

### Summary Statistics and Heritabilities

Mean phenotypic values for height, tiller number, panicle length, proportion of spikelets filled, yield per plant, and potential yield were respectively 85 cm, 14 tillers, 226 mm, 0.757, 36 g per plant, and 54 g per plant (Supplementary Figure 1; Table 1). Highest entry-mean heritability observed was for height at 0.906, and lowest entry-mean heritability observed was for potential yield at 0.311 (Table 2). Narrow-sense heritabilities on a single-plant basis were greatest for the proportion of spikelets filled at 0.864 and least for potential yield at 0.271 (Table 2). Because the narrow-sense heritability of proportion of spikelets filled was high, perhaps due to differing genetic architectures between TGMS and CMS lines for the trait, we also estimated the narrow-sense heritability in the CMS lines only as 0.922 by removing observations of the TGMS line A07 from the model. Principal components analysis of the parental genotypes showed clustering of the males and females, but divergence of the male and female parents was not due to historical reciprocal recurrent selection (Supplementary Figure 2).

**TABLE 1:** Trait mean phenotypic values and standard deviations overall and within environments for the F_1_ hybrids.

Trait	Mean ± Standard Deviation, Overall	Mean ± Standard Deviation, Irrigated Lowland	Mean ± Standard Deviation, Irrigated Upland
Height (cm)	85 ± 15	89 ± 13	70 ± 11
Tiller Number	14 ± 5	16 ± 4	10 ± 3
Panicle Length (mm)	226 ± 24	231 ± 22	213 ± 25
Proportion of Spikelets Filled^∗^		0.757 ± 0.149	
Yield per Plant (g)	36 ± 19	44 ± 17	18 ± 7
Potential Yield^∗^ (g)		54 ± 20	

*^∗^Proportion of spikelets filled and potential yield were only phenotyped in the irrigated lowland environment.*

**TABLE 2:** Estimates and their standard errors for each trait of entry-mean heritability of the F_1_ hybrids and narrow-sense heritability on a single-plant basis.

Trait	Entry-Mean Heritability	Narrow-Sense Heritability
Height	0.906 ± 0.007	0.719 ± 0.029
Tiller Number	0.505 ± 0.034	0.376 ± 0.054
Panicle Length	0.824 ± 0.014	0.542 ± 0.046
Proportion of Spikelets Filled	0.780 ± 0.014	0.864 ± 0.014*
Yield per Plant	0.433 ± 0.038	0.328 ± 0.055
Potential Yield	0.311 ± 0.046	0.271 ± 0.063

*^∗^In the CMS lines only, the narrow-sense heritability for proportion of spikelets filled was 0.922 ± 0.008.*

### Model Fit, Predictive Ability, and Prediction Accuracy

For all traits, model fit was superior for the genomic GCA + SCA model compared to the genomic GCA model as assessed by either AIC or BIC (Supplementary Tables 2, 3). However, the predictive abilities and accuracies of the genomic GCA + SCA models were not substantially different from the genomic GCA models (Supplementary Tables 4, 5; Tables 3, 4). Mean prediction accuracies for male GCA ranged from 0.215 to 0.318 for the genomic GCA models and 0.233 to 0.332 in the genomic GCA + SCA models (Table 4). Prediction accuracies for untested females were not estimated. For hybrids in the T0 set, mean genomic GCA model accuracies ranged 0.039 to 0.394, while the genomic GCA + SCA model accuracies ranged from 0.043 to 0.490. For hybrids in the T1 set, mean genomic GCA model accuracies ranged from 0.476 to 0.806, while the genomic GCA + SCA model accuracies ranged from 0.509 to 0.827. For hybrids in the T1F set, mean genomic GCA model accuracies ranged from 0.310 to 0.908, while the genomic GCA + SCA model accuracies ranged from 0.364 to 0.943. For hybrids in the T1M set, mean genomic GCA model accuracies ranged from 0.537 to 0.742, while the genomic GCA + SCA model accuracies ranged from 0.423 to 0.785. For hybrids in the T2 set, mean genomic GCA model accuracies ranged from 0.769 to 0.948, while the genomic GCA + SCA model accuracies ranged from 0.772 to 0.956 (Table 3). However, in the hybrid T0 case, accuracy for yield per plant was increased from 0.215 to 0.490 by inclusion of the SCA effect. No other trait had more than a 10% increase in accuracy by inclusion of the SCA effect in the T0 case, and substantial increases in accuracy with inclusion of the SCA effect were not observed for yield per plant in scenarios besides T0. Except in the case of proportion of spikelets filled, accuracy in the T1F scenarios was always substantially higher than the T1M scenarios.

**TABLE 3:** Mean prediction accuracies ± standard error thereof in cross-validation of the genomic prediction models for hybrids.

Trait	T0	T1	T1F	T1M	T2
***Genomic GCA model***					
Height	0.345 ± 0.013	0.793 ± 0.009	0.908 ± 0.007	0.562 ± 0.006	0.948 ± 0.003
Tiller Number	0.162 ± 0.005	0.628 ± 0.014	0.644 ± 0.015	0.537 ± 0.008	0.812 ± 0.007
Panicle Length	0.394 ± 0.012	0.806 ± 0.009	0.906 ± 0.008	0.574 ± 0.005	0.942 ± 0.005
Proportion of Spikelets Filled	0.039 ± 0.003	0.476 ± 0.003	0.310 ± 0.006	0.742 ± 0.003	0.769 ± 0.003
Yield per Plant	0.215 ± 0.012	0.688 ± 0.008	0.741 ± 0.008	0.646 ± 0.007	0.820 ± 0.005
Potential Yield	0.380 ± 0.015	0.719 ± 0.011	0.842 ± 0.009	0.566 ± 0.011	0.855 ± 0.006
***Genomic GCA* + *SCA model***					
Height	0.414 ± 0.019	0.820 ± 0.010	0.943 ± 0.008	0.498 ± 0.014	0.956 ± 0.005
Tiller Number	0.190 ± 0.007	0.640 ± 0.016	0.676 ± 0.016	0.442 ± 0.012	0.772 ± 0.013
Panicle Length	0.446 ± 0.020	0.827 ± 0.011	0.940 ± 0.011	0.423 ± 0.018	0.928 ± 0.011
Proportion of Spikelets Filled	0.043 ± 0.003	0.509 ± 0.004	0.364 ± 0.007	0.785 ± 0.005	0.809 ± 0.004
Yield per Plant	0.490 ± 0.022	0.715 ± 0.009	0.795 ± 0.009	0.532 ± 0.014	0.822 ± 0.006
Potential Yield	0.415 ± 0.016	0.723 ± 0.012	0.847 ± 0.010	0.525 ± 0.014	0.851 ± 0.007

*For each of the 500 cross-validation folds, 4 female parents and 127 male parents were chosen to have hybrid progeny included in the training set. The total number of hybrid genotypes sampled for training was 150. Accuracies are reported for hybrids which were not included in the training set which had neither parent in the training set (T0), one parent in the training set (T1), the female parent only in the training set (T1F), the male parent only in the training set (T1M), and both parents in the training set (T2).*

**TABLE 4:** Model prediction accuracy and standard error for male GCA for each trait as estimated by ten-fold cross-validation.

Trait	Genomic GCA model	Genomic GCA + SCA model
Height	0.232 ± 0.056	0.233 ± 0.067
Tiller Number	0.215 ± 0.060	0.261 ± 0.065
Panicle Length	0.224 ± 0.043	0.269 ± 0.046
Proportion of Spikelets Filled	0.318 ± 0.083	0.332 ± 0.079
Yield per Plant	0.219 ± 0.072	0.241 ± 0.079
Potential Yield	0.292 ± 0.078	0.233 ± 0.096

The accuracy of phenotypic selection for hybrid genetic value ranged from 0.566 to 0.952 among traits (Table 5). For the GCA model, the accuracy of phenotypic selection for male GCA ranged from 0.484 to 0.861, and the accuracy of phenotypic selection for female GCA ranged from 0.853 to 0.910 (Tables 6, 7; Supplementary Tables 6, 7). For the GCA + SCA model, the accuracy of phenotypic selection for male GCA ranged from 0.253 to 0.798, and the accuracy of phenotypic selection for female GCA ranged from 0.850 to 0.910 (Tables 6, 7; Supplementary Tables 6, 7).

**TABLE 5:** Reliabilities and accuracies of phenotypic selection for hybrid performance.

Trait	Reliability	Accuracy
Height	0.906	0.952
Tiller Number	0.533	0.730
Panicle Length	0.828	0.910
Proportion of Spikelets Filled	0.791	0.889
Yield per Plant	0.466	0.682
Potential Yield	0.321	0.566

**TABLE 6:** Accuracies of phenotypic selection for male GCA.

Trait	GCA model	GCA + SCA model
Height	0.809	0.627
Tiller Number	0.644	0.570
Panicle Length	0.680	0.253
Proportion of Spikelets Filled	0.861	0.798
Yield per Plant	0.640	0.562
Potential Yield	0.484	0.454

**TABLE 7:** Accuracies of phenotypic selection for female GCA.

Trait	GCA model	GCA + SCA model
Height	0.906	0.906
Tiller Number	0.860	0.858
Panicle Length	0.910	0.910
Proportion of Spikelets Filled	0.898	0.876
Yield per Plant	0.853	0.850
Potential Yield	0.900	0.899

### Hybrid Genetic Value and Parental GCA

The genomic GCA + SCA model was used to rank hybrid genetic values. The maximum predicted F_1_ hybrid yield was 43.352 grams per plant, which scaled to 8.670 tons per hectare (Supplementary Table 8). The maximum predicted F_1_ hybrid potential yield was 77.401 grams per plant, scaling to 15.479 tons per hectare (Supplementary Table 9). Over half of the top 20 F_1_ hybrids in terms of yield per plant had phenotypic observations available, though the top-ranked hybrid did not. The relative performance of the F_1_ hybrids compared to the commercial inbred checks (commercial heterosis) ranged from −43.9% to 70.0% for yield per plant (Supplementary Figure 3; Table 8). The maximum genomic predicted GCA for yield per plant in females and males respectively were 36.341 and 34.047; both of the female and male lines top-ranked for GCA had phenotypic observations available (Supplementary Tables 10, 11).

**TABLE 8:** Mean, standard deviation, and range of relative yield per plant of the F_1_ hybrids compared to each inbred check.

Check	Mean Relative Performance ± SD	Range
WTR-1	0.206 ± 0.143	−0.238—0.679
Y 134 (DP 6)	0.191 ± 0.141	−0.248—0.659
Khazar (DP 8)	0.283 ± 0.147	−0.218—0.724
OM 997 (DP 10)	0.283 ± 0.152	−0.190—0.786
M 401 (DP 17)	0.179 ± 0.140	−0.256—0.642
X 21 (DP 19)	0.289 ± 0.153	−0.186—0.796

### Mid-Parent Heterosis and Parental SDAF

**FIGURE 2:**
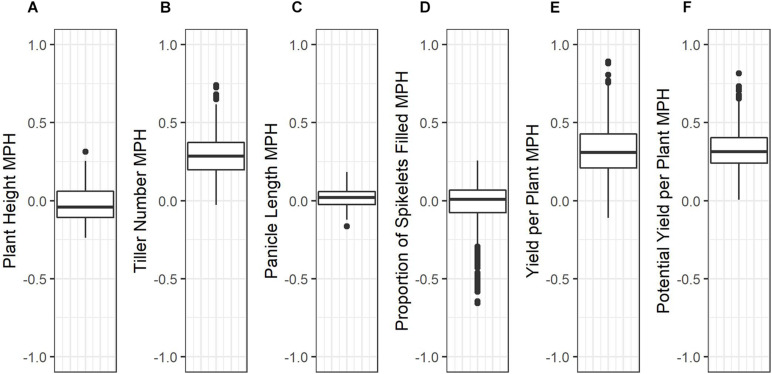
Box plots of mid-parent heterosis for each trait in the phenotyped F_1_ hybrids. **(A)** Plant height (cm). **(B)** Tiller number. **(C)** Panicle length (mm). **(D)** Proportions of spikelets filled. **(E)** Yield per plant (g). **(F)** Potential yield per plant (g).

**FIGURE 3:**
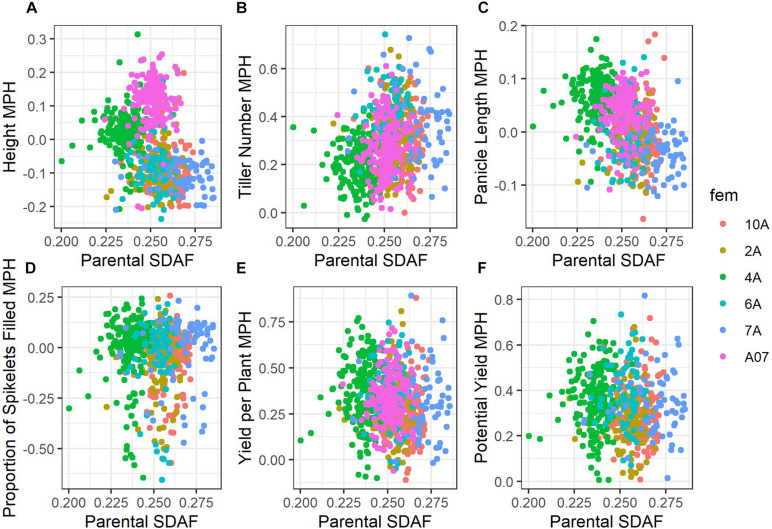
Scatterplots of mid-parent heterosis against parental SDAF. Points are colored according to female parent. **(A)** Height. **(B)** Tiller number. **(C)** Panicle length. **(D)** Proportion grains filled. **(E)** Yield per plant. **(F)** Potential yield.

Average mid-parent heterosis was positive, though not extremely so, for all traits except height and proportion of spikelets filled (Figure 2 and Table 9). Yield per plant and its component trait, tiller number, showed the highest average heterosis; average heterosis for yield was 32.0%. Parental SDAF ranged from 0.200 to 0.285 in the phenotyped F_1_ hybrids (Supplementary Figure 4). Overall, in all hybrids, parental SDAF was significantly correlated with mid-parent heterosis for all traits except proportion of spikelets filled (Figure 3 and Table 10). Interestingly, the direction of the correlation was negative for all traits except tiller number. The strongest correlation of mid-parent heterosis and SDAF for hybrids overall was for panicle length. However, when hybrids were grouped into families by female parent, there were no significant correlations between parental SDAF and mid-parent heterosis.

**TABLE 9:** Mean, standard deviation, and range of mid-parent heterosis for each trait.

Trait	Mean Mid-Parent Heterosis ± SD	Range
Height	−0.019 ± 0.106	−0.237—0.313
Tiller Number	0.292 ± 0.132	−0.026—0.741
Panicle Length	0.017 ± 0.054	−0.163—0.183
Proportion of Spikelets Filled	−0.034 ± 0.160	−0.657—0.258
Yield per Plant	0.320 ± 0.163	−0.110—0.892
Potential Yield	0.327 ± 0.131	0.005—0.817

**TABLE 10:** Pearson’s correlation coefficient of mid-parent heterosis and SDAF with 95% confidence intervals of the coefficient and *t*-tests of significance conducted at α = 0.05.

Trait	*r* ± 95% CI	*t*	*df*	*P*
**Height**				
*Overall*	−0.3300.062	–9.791	783	< 0.001
*10A*	−0.0410.157	–0.512	154	0.610
*2A*	0.0070.176	0.077	122	0.939
*4A*	0.0310.145	0.419	181	0.676
*6A*	−0.0440.227	–0.381	73	0.705
*7A*	−0.0730.237	–0.592	66	0.556
*A07*	0.0840.146	1.118	177	0.265
**Tiller Number**				
*Overall*	0.2990.064	8.725	778	< 0.001
*10A*	−0.1430.155	–1.786	152	0.076
*2A*	0.0580.176	0.644	121	0.521
*4A*	0.0270.145	0.357	180	0.722
*6A*	0.0320.227	0.269	73	0.788
*7A*	−0.0840.239	–0.677	65	0.501
*A07*	0.0450.146	0.601	177	0.549
**Panicle Length**				
*Overall*	−0.4300.057	–13.309	783	< 0.001
*10A*	−0.0420.157	–0.516	154	0.607
*2A*	−0.0840.175	–0.935	122	0.352
*4A*	0.0810.144	1.094	181	0.275
*6A*	0.0660.226	0.568	73	0.572
*7A*	0.0130.238	0.103	66	0.918
*A07*	0.0580.146	0.772	177	0.441
**Proportion of Spikelets Filled**				
*Overall*	−0.0150.081	–0.363	588	0.717
*10A*	0.1270.156	1.578	151	0.117
*2A*	0.0630.177	0.687	120	0.493
*4A*	0.1030.146	1.364	174	0.174
*6A*	0.0270.228	0.23	72	0.818
*7A*	0.0830.242	0.663	63	0.510
**Yield per Plant**				
*Overall*	−0.1310.069	–3.698	781	< 0.001
*10A*	0.0140.157	0.169	154	0.866
*2A*	0.0410.176	0.450	122	0.653
*4A*	0.1160.144	1.562	180	0.120
*6A*	0.0400.227	0.346	73	0.730
*7A*	−0.0220.238	–0.176	66	0.861
*A07*	0.1040.146	1.389	176	0.167
**Potential Yield**				
*Overall*	−0.0920.08	–2.225	585	0.026
*10A*	−0.0310.159	–0.386	151	0.700
*2A*	−0.0050.178	–0.059	120	0.953
*4A*	0.0870.148	1.141	171	0.255
*6A*	0.0420.228	0.360	72	0.720
*7A*	−0.0080.244	–0.060	63	0.952

*Overall correlations as well as correlations within female families are reported. Correlations are not available within the A07 female family for potential yield or proportion of spikelets filled because phenotypic observations of the A07 line were not available at the location in which the traits were phenotyped, irrigated lowland.*

## Discussion

The objectives of this study were (1) to identify high-yielding F_1_ hybrids from crosses of IRRI male sterile lines with stress-tolerant male lines, (2) to identify parental lines with high GCA for use in future reciprocal recurrent genomic selection programs, and (3) to evaluate genomic prediction and phenotypic selection accuracies in our hybrid breeding population, with the end goal of developing stress-tolerant hybrid rice varieties. Compared to inbred commercial checks (which were also progenitors of the male lines), phenotyped F_1_ hybrids showed genetic yield advantages of up to 80% and warrant further testing (Supplementary Figure 3; Table 8). Although the genetic yield of the top-performing F_1_ hybrid observed in the study environment was 8.670 tons per hectare, this measure pertains to the study environment only—which included both standard irrigated conditions and stressful upland conditions—and only the plant population densities used, which were lower than those observed in farmers’ fields (Supplementary Table 8). Relative to the mid-parent, the F_1_ hybrids showed on average 32.0% mid-parent heterosis for yield, which is consistent with literature averages of 10 to 30% in rice (Figure 2 and Table 9; Janaiah and Xie, 2010; Longin et al., 2012; Spielman et al., 2013). However, the maximum mid-parent heterosis observed for yield was 89.2%, and heterosis up to 48.3% was observed within a single standard deviation of the mean (Figure 2 and Table 9). Because heterosis is present in the F_1_ hybrids and SCA as well as GCA variance was detected, recurrent selection for GCA in the male and female lines tested should allow development of heterotic pools. However, we did not evaluate the relative efficiency of hybrid vs. line breeding for our population given the estimated GCA and SCA variance detected in our population or other relevant factors, and further investigation of this topic is warranted.

### Fertility Restoration and Genetic Architecture of Spikelet Filling

Interestingly, narrow-sense heritability for the proportion of spikelets filled was relatively high at 0.600 (Table 2). Although variation in grain fill is generally driven by environmental factors in rice, with starch synthesis and deposition depending on available assimilate, in rice hybrids absence of grain fill can be due to spikelet sterility from lack of fertility-restoring (*Rf*) alleles (Peng et al., 1998; Tang et al., 2017). Because fertility restoration is only relevant in hybrids of CMS parents, not TGMS parents, we estimated narrow-sense heritability for proportion of spikelets filled in the CMS-derived hybrids only as 0.922 (Table 2). The relatively high proportion of phenotypic variance explained by additive genetic variance for this trait in CMS lines suggests that segregation for fertility restoration played a role in the proportion of spikelets filled in the CMS-derived hybrids and as such in observed yield per plant. Concordantly, selection accuracy for proportion of spikelets filled was substantially higher in the T1M hybrids than T1F hybrids in cross-validation, though T1F hybrids had higher accuracies than T1M for all other traits. Screening the population for known major fertility restoration alleles at *Rf3* and *Rf4* may allow the use of marker-assisted selection to improve selection accuracy (Tang et al., 2017). It may also be possible to select for fertility restoration by genomic prediction rather than mapping fertility-restoring alleles of more minor or modifying effect. Selection for fertility restoration may effectively unlock observed yield potential in future hybrids and fix *Rf* alleles in the male heterotic pool.

### Accuracies of Genomic Prediction for Hybrid Genetic Value and Parent GCA

Genomic prediction model accuracies were high in unobserved T2 F_1_ hybrids, for which both parents were included in the training set (Table 3). All F_1_ hybrids surveyed were closely related; closely related individuals have smaller effective population size, which reduces the effective number of loci controlling traits and is expected to increase prediction accuracy (Supplementary Figure 5; Daetwyler et al., 2010). Accuracy appeared to be driven primarily by estimation of the female line effects, and accuracy in T2 hybrids was not substantially different from T1F hybrids (Table 3). The exception was proportion of spikelets filled, for which the male (restorer) line effects were more relevant. As expected, accuracy in the T0 hybrids was low, though positive and improved substantially for yield per plant by the inclusion of SCA effects in the model (Table 3).

Accuracy was low for genomic estimated male GCA (< 0.300) despite that the males all shared a recurrent parent and as such a large proportion of their genomes (75% ± Mendelian sampling and selection; Supplementary Figure 6; Table 4). Although the male donor progenitors were diverse, multiple male lines per donor were sampled. Low accuracies of genomic predictions of male GCA may have been due to highly unbalanced crossing of males to females, with no single male crossed to all females. It was not possible to estimate accuracy for genomic estimated GCA in the females, which were also closely related but more extensively phenotyped (Supplementary Figure 7).

Phenotypic accuracies were similar to or lower than genomic prediction accuracies for hybrid performance in the T2 case (Table 5; Rutkoski et al., 2015). Notably, accuracy of genomic prediction of hybrid yield (0.820) was greater than accuracy of the phenotypes (0.682). However, for male GCA, the phenotypic accuracies greatly exceeded those of genomic prediction for both the GCA and the GCA + SCA models (Table 6). It was not possible to compare genomic and phenotypic selection accuracies for female GCA.

### Inconsistent Correlations of Mid-Parent Heterosis and Parental SDAF

Considering all hybrids in the study, we observed parental SDAF to be negatively correlated with mid-parent heterosis and hybrid genetic value for all traits surveyed except tiller number, for which SDAF was positively correlated with mid-parent heterosis (Figure 3 and Table 10). In many species, parental SDAF (or other measures of genetic distance) is positively correlated with heterosis due to release from inbreeding depression to a point, as dominant alleles mask deleterious recessive alleles in hybrids (Falconer and Mackay, 1996). However, as genetic distance increases, outbreeding depression eventually prevails as favorable epistatic combinations of genes are separated (Lynch and Walsh, 1998). A common manifestation of outbreeding depression is fertility barriers (Edmands, 2002). In the case of yield per plant, we cannot eliminate the possibility that genetic distance is correlated with absence of wide-cross compatibility alleles known to affect seed set, given the inter-subspecific diversity present in the male lines (Ji et al., 2005). However, given the intense selection on the male lines, it seems possible that wide-cross compatibility in the males may have also been positively and indirectly selected with yield. Genetic distance could also be correlated with absence of fertility restoring alleles by chance. However, yield potential corrects for fertility restoration by estimating yield as if all spikelets were filled to the average weight observed in the study, and overall mid-parent heterosis for yield potential was also negatively correlated with parental SDAF. Importantly, though unsurprisingly given the relationships of the BC_1_F_5_ male lines, the correlation of parental SDAF and mid-parent heterosis was not observed within female families of hybrids (Table 10). This suggests that the negative correlations observed in hybrids overall were due to differences in female genetic distance from the average male. More crucially for practical purposes, whether genetic distance is indicative of mid-parent heterosis depends on the population defined, even in closely related hybrids.

### Future Directions for IRRI Hybrid Rice Breeding

Based on the study findings, we caution against the conventional wisdom that increased genetic distance between parents alone will always confer improved hybrid performance or positive heterosis. Increased genetic distance in the potential founders of heterotic pools of rice screened was not reliably associated with desired positive heterosis for yield, even though the pools could be genetically distinguished. In this population, and probably in rice more generally, empirical selection for GCA is preferable to selection based on genetic distance to breed high-performing hybrid rice.

For hybrid performance, genomic prediction accuracies were similar to or higher than phenotypic accuracies. Therefore, genomic prediction could be useful for product development in the population of study. Most notably, inclusion of genomic information increased prediction accuracy for hybrid yield per plant by approximately 13.8% compared to phenotype alone (Tables 3, 5). Observed accuracies of prediction of unobserved hybrids with at least one parent in the training population (T1) were also positive and substantial, suggesting that on average genomic prediction could allow identification of further crosses with high value in the population of study. Genomic prediction accuracies for hybrids with neither parent observed (T0) were not as high as in the T1 case, but were nonetheless positive.

In contrast, genomic prediction accuracies for male GCA were substantially lower than phenotypic accuracies. For yield, genomic prediction accuracies were approximately three times less than phenotypic selection accuracies. However, reciprocal recurrent genomic selection for GCA can reduce cycle length by two-thirds compared to reciprocal recurrent phenotypic selection, because parents can be immediately recycled using genomic predictions of their GCA, leading to a cycle length of one (Powell et al., 2020). In conventional reciprocal recurrent selection, it is necessary to cross new parents to the opposing pool and phenotype the inter-pool crosses to estimate GCA before intra-pool recycling is possible, which increases the cycle length to three (Rembe et al., 2019). The genomic prediction accuracies observed in the study would provide comparable genetic gain to phenotypic selection if used to reduce the breeding cycle length to one, assuming that reduction in cycle length has no effect on genomic prediction accuracy and that accuracy of genomic prediction of female GCA (which could not be estimated, but for which more observations per female were available) is the same or higher than male GCA (Rembe et al., 2019; Powell et al., 2020). Increases in accuracies of genomic prediction of GCA relative to phenotypic selection are likely possible, as the training set of related hybrids would build over time in a closed population, and more complete and informative crossing designs could provide phenotypes. The potential of hybrid breeding strategies in IRRI germplasm would benefit from further assessment by simulation (Faux et al., 2016; Gaynor et al., 2020).

## Data Availability Statement

The phenotypic datasets generated and analyzed for this study can be found in Supplementary File 1. The raw genotype data used in this study can be found at NCBI under accession PRJNA479931, excluding the female genotype data. The female genotype data for this article are not publicly available as they are the property of the International Rice Research Institute. Requests to access the female genotype data should be directed to JA at j.ali@irri.org.

## Author Contributions

ML planned the study, executed the field trial and analysis, and wrote the manuscript. JA, MA, EA, MP, and MS developed plant materials used. EA, MP, and MS directed field operations. AL supervised the statistical analysis. AS supervised the marker analysis. JR supervised the quantitative genetic analysis. JA conceived of the study, directed the study, and provided breeding insights. All authors edited and approved the final manuscript.

## Conflict of Interest

The authors declare that the research was conducted in the absence of any commercial or financial relationships that could be construed as a potential conflict of interest.
