# Supplementary material for: Genomic Prediction of Yield Traits in Single-Cross Hybrid Rice (Oryza sativa L.)
Source: Front Genet. 2021 Jun 30;12:692870. doi: 10.3389/fgene.2021.692870 (PMC8278103; doi:10.3389/fgene.2021.692870)
Supplement: Supplementary file 1 [file Data_Sheet_1.docx]

**
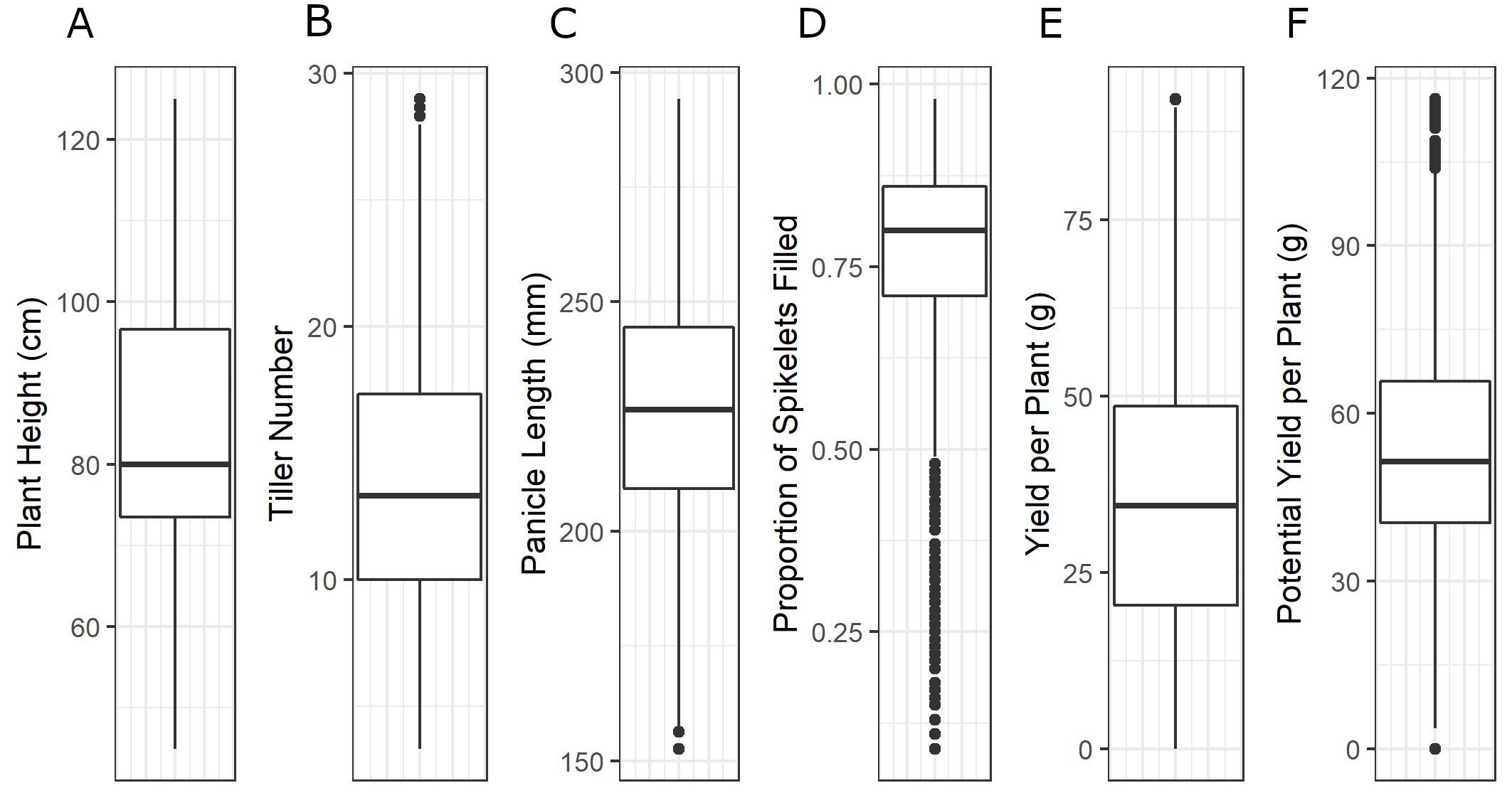
**

**Fig. S1** Box plots of trait raw phenotypes in the F_1_ hybrids. A) Plant height (cm). B) Tiller number. C) Panicle length (mm). D) Proportions of spikelets filled. E) Yield per plant (g). F) Potential yield per plant (g).


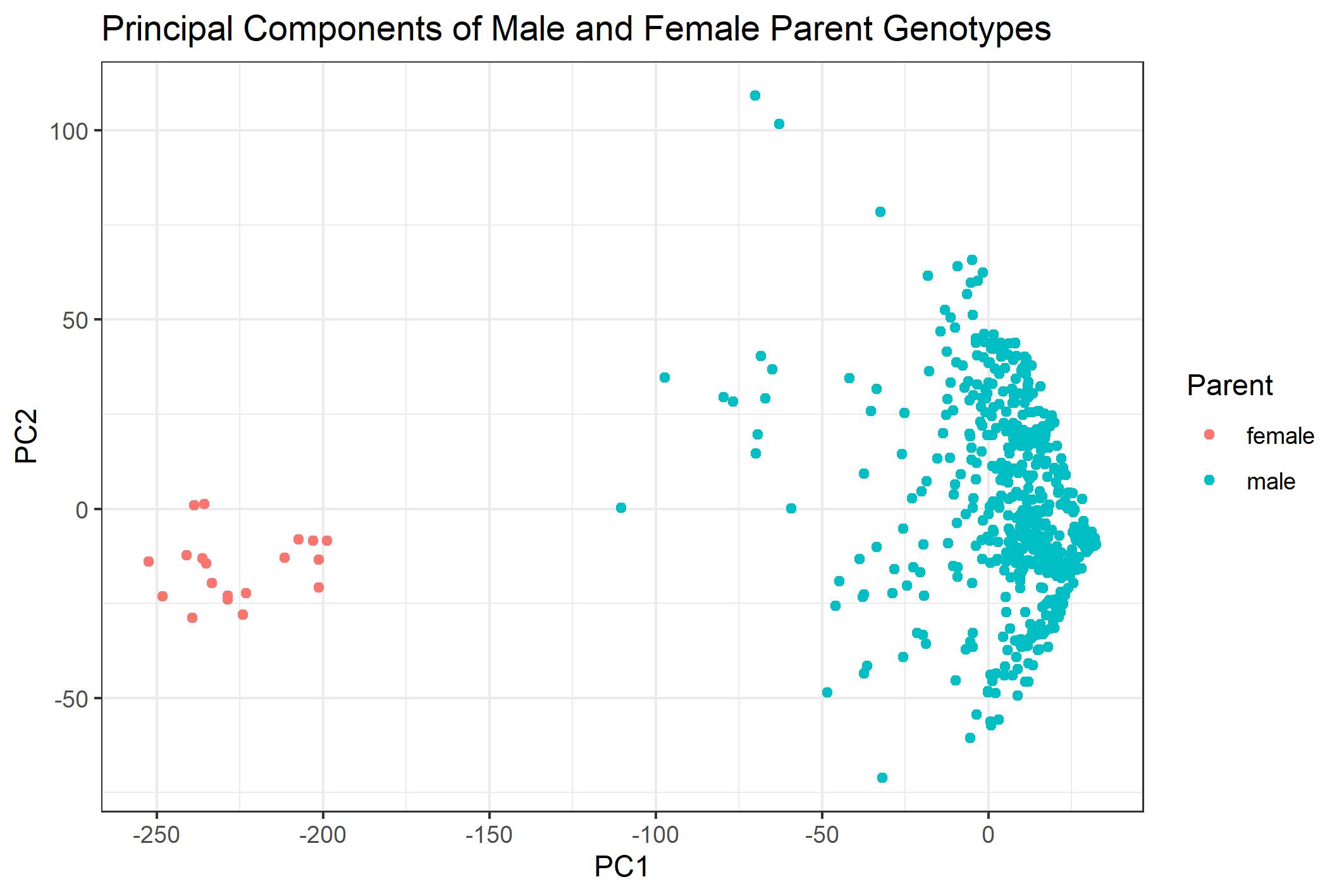


**Fig. S2** Plot of first two principal components in the parental genotypes. The females, shown in red, cluster separately from the males, shown in blue. The first principal component (PC1) explained 10.4% of the variance, and the second principal component (PC2) explained 3.1% of the variance.

**
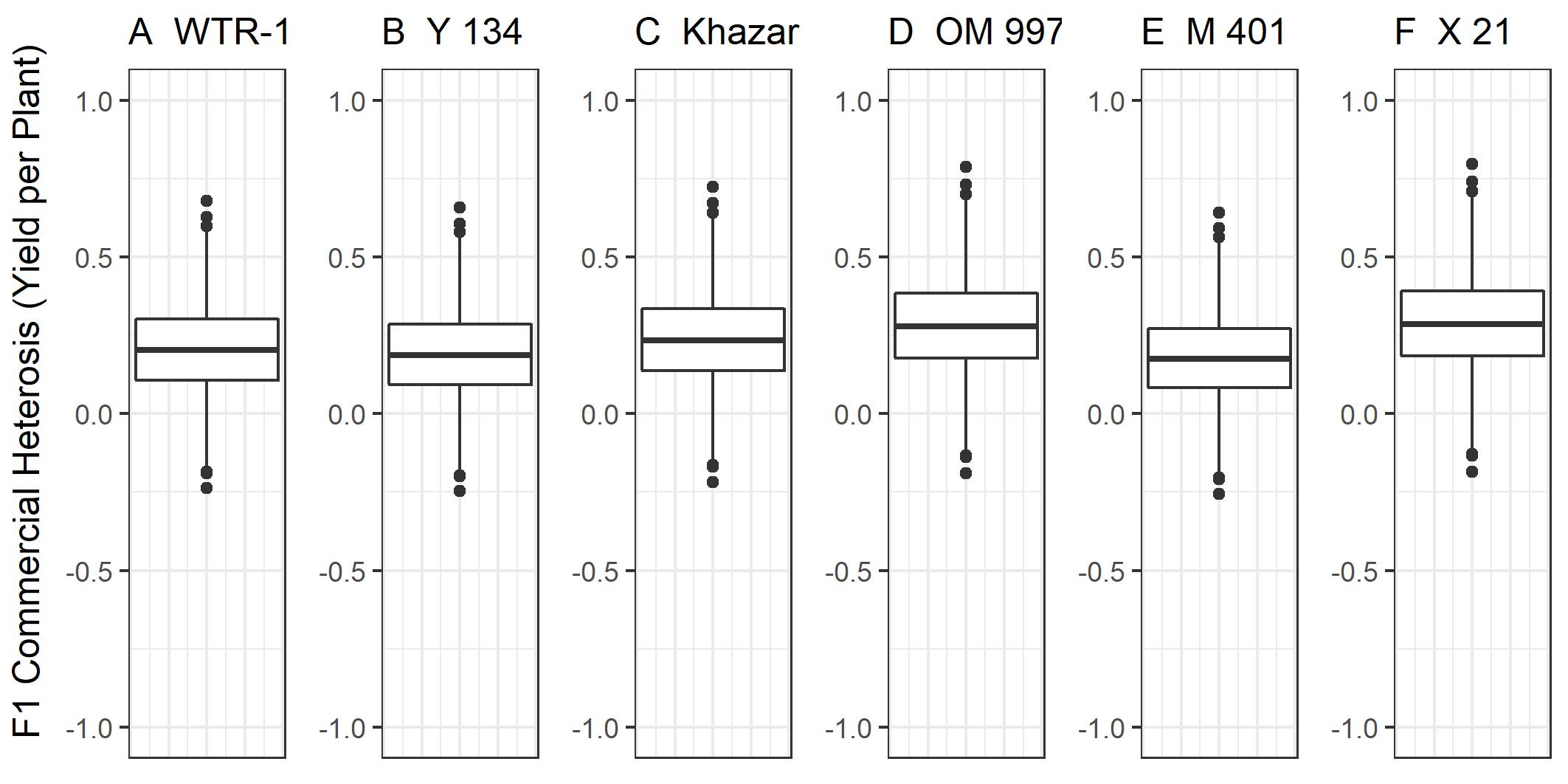
**

**Fig. S3** F_1_ hybrid relative performance over the checks (commercial heterosis). Only lines with phenotypic records available displayed.

**
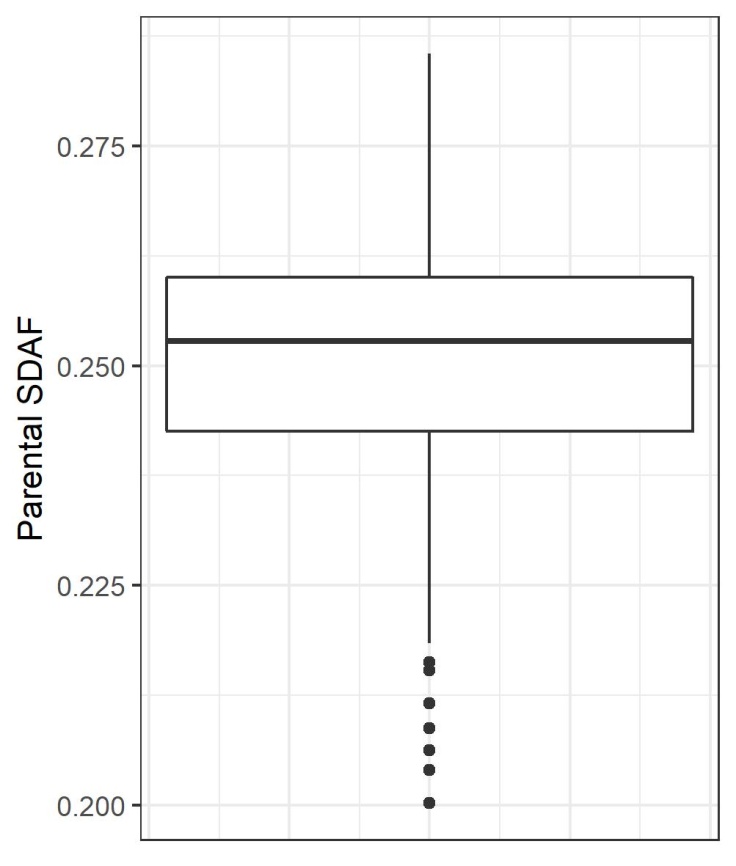
**

**Fig. S4** Box plot of parental SDAF in the F_1_ hybrids.


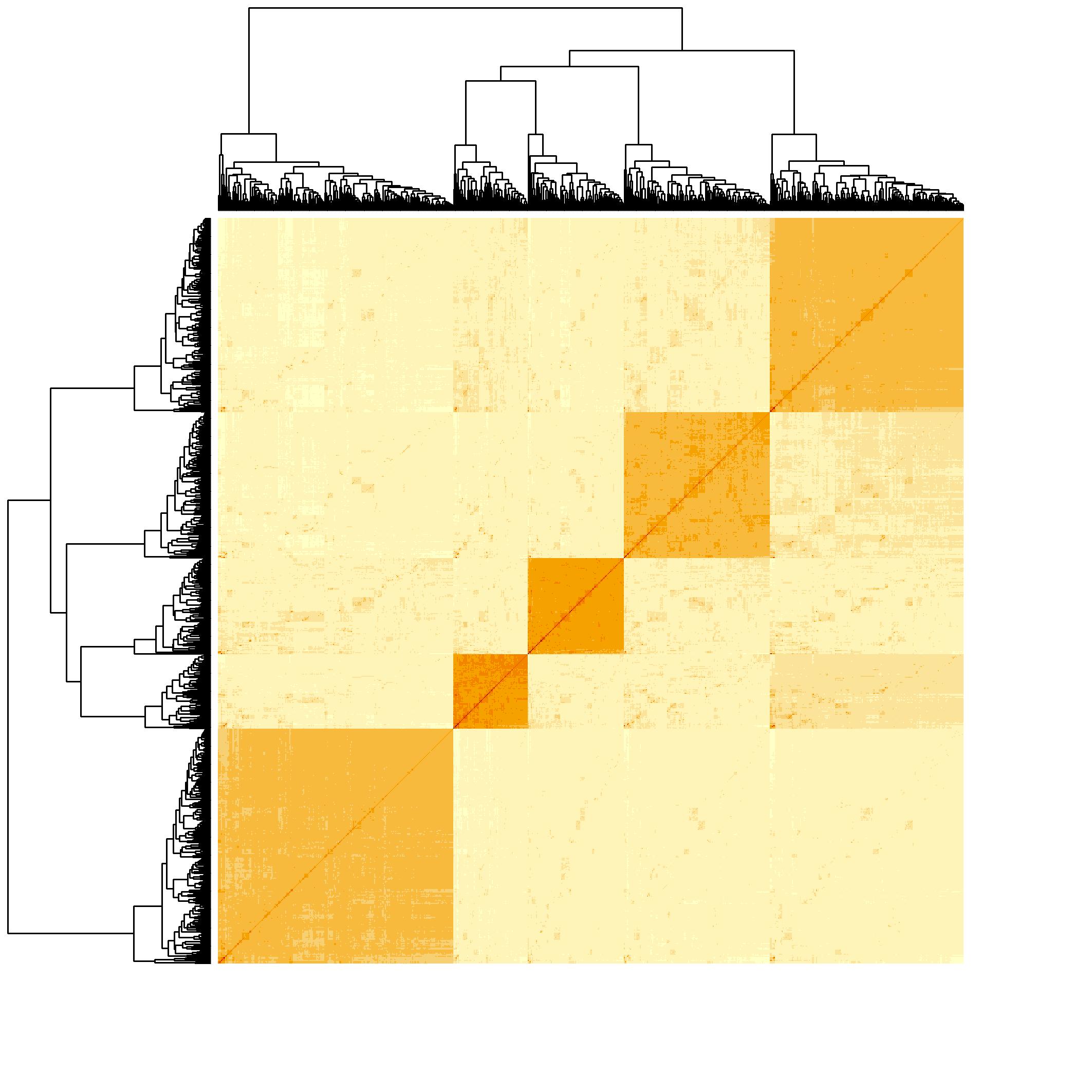


**Fig. S5** Heatmap of the F_1_ hybrids’ additive genomic relationship matrix.


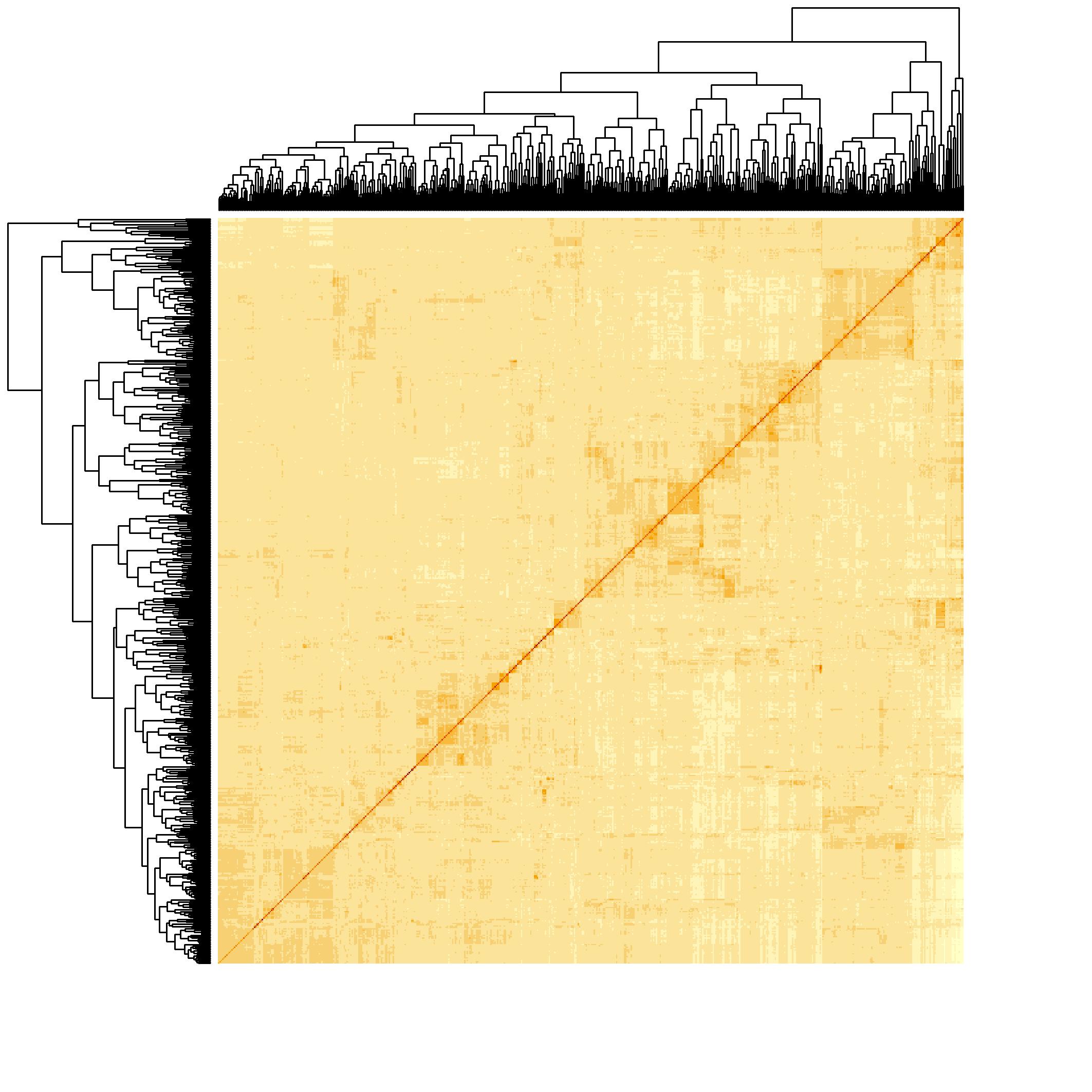

**Fig. S6** Heatmap of the male parents’ additive genomic relationship matrix.


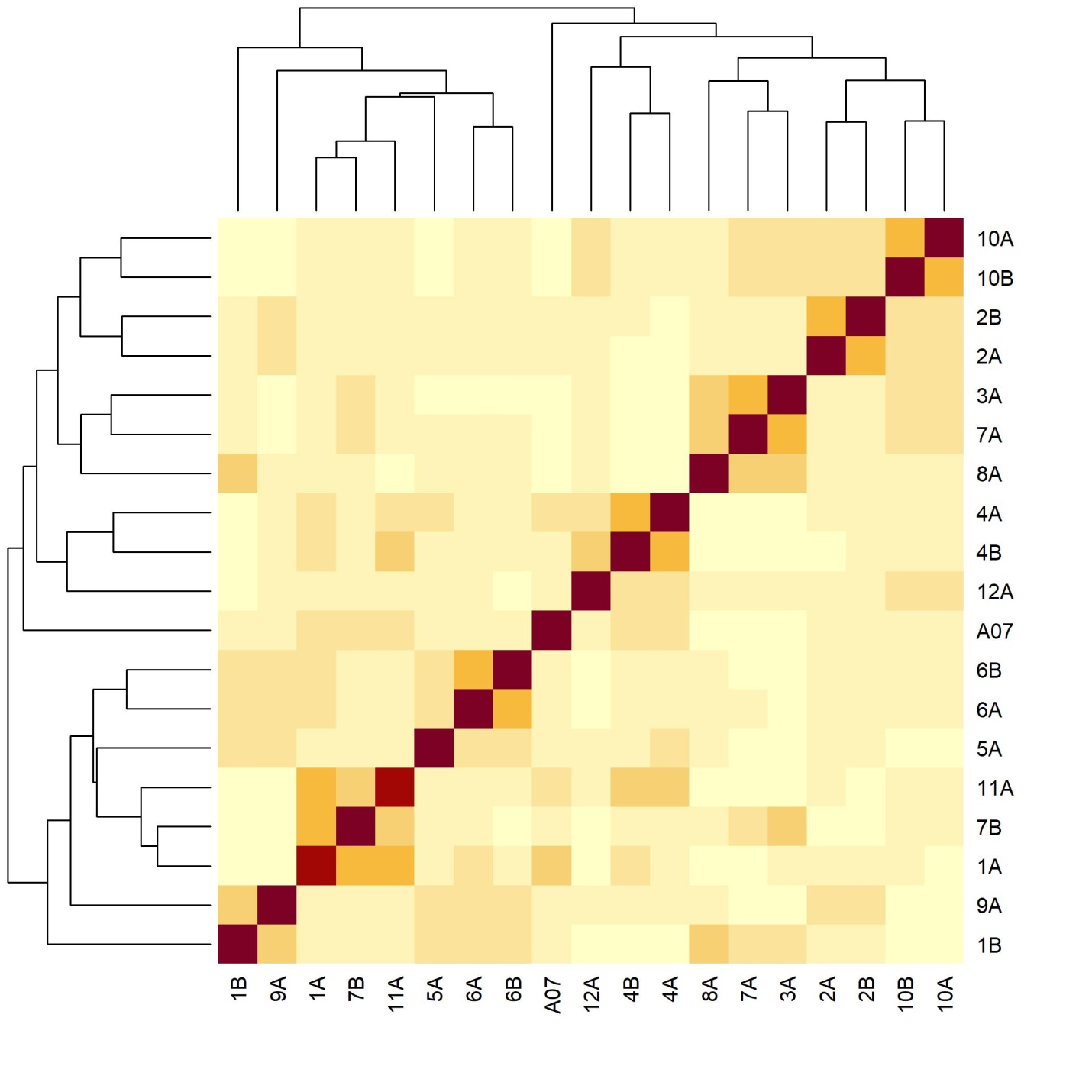


**Fig. S7** Heatmap of the female parents’ and female restorer lines’ additive genomic relationship matrix.
