# Supplementary material for: Genomic Prediction of Yield Traits in Single-Cross Hybrid Rice (Oryza sativa L.)
Source: Front Genet. 2021 Jun 30;12:692870. doi: 10.3389/fgene.2021.692870 (PMC8278103; doi:10.3389/fgene.2021.692870)
Supplement: Supplementary file 3 [file Data_Sheet_3.docx]

**Table S1** Female lines used for crossing are indicated in the row and column headers. In a given row and column, the number of male lines crossed to both females in the row and column header is provided, i.e. the pairwise overlap of females in male lines crossed. The diagonal contains the number of male line crossed to the given single female.

| **Female** | 10A | 2A | 4A | 6A | 7A | A07 |
| --- | --- | --- | --- | --- | --- | --- |
| 10A | 194 | 82 | 97 | 56 | 39 | 107 |
| 2A |  | 146 | 79 | 15 | 50 | 84 |
| 4A |  |  | 235 | 61 | 21 | 105 |
| 6A |  |  |  | 96 | 1 | 50 |
| 7A |  |  |  |  | 75 | 43 |
| A07 |  |  |  |  |  | 192 |

**Table S2** Prediction model AIC for each trait.

| **Trait** | **GCA** | **GCA + SCA** | **Genomic GCA** | **Genomic GCA + SCA** |
| --- | --- | --- | --- | --- |
| Height | -1993.074 | -2140.428 | -1994.448 | -2122.584 |
| Tiller Number | 780.001 | 767.676 | 772.809 | 761.934 |
| Panicle Length | -209.071 | -312.758 | -219.971 | -312.816 |
| Proportion of Spikelets Filled | 956.326 | 831.030 | 921.973 | 807.592 |
| Yield per Plant | 647.750 | 637.354 | 645.734 | 633.112 |
| Potential Yield | 1276.518 | 1275.510 | 1271.489 | 1266.205 |

**Table S3** Prediction model BIC for each trait.

| **Trait** | **GCA** | **GCA + SCA** | **Genomic GCA** | **Genomic GCA + SCA** |
| --- | --- | --- | --- | --- |
| Height | -1987.367 | -2134.721 | -1988.741 | -2116.878 |
| Tiller Number | 785.670 | 773.374 | 778.508 | 767.633 |
| Panicle Length | -203.367 | -307.053 | -214.266 | -307.112 |
| Proportion of Spikelets Filled | 961.673 | 836.377 | 927.320 | 812.938 |
| Yield per Plant | 653.450 | 643.054 | 651.433 | 638.812 |
| Potential Yield | 1281.858 | 1280.851 | 1276.829 | 1271.546 |

**Table S4** Mean predictive ability ± standard error thereof in cross-validation of the genomic prediction models for hybrids. For each of the 500 cross-validation folds, 4 female parents and 127 male parents were chosen to have hybrid progeny included in the training set. The total number of hybrid genotypes sampled for training was 150. Abilities are reported for hybrids which were not included in the training set which had neither parent in the training set (T0), one parent in the training set (T1), the female parent only in the training set (T1F), the male parent only in the training set (T1M), and both parents in the training set (T2). The number in parentheses indicates the number of hybrids in each category averaged across cross-validation folds.

| **Trait** | **T0** | **T1** | **T1F** | **T1M** | **T2** |
| --- | --- | --- | --- | --- | --- |
| *Genomic GCA model* |  |  |  |  |  |
| Height | 0.344 ± 0.013 (460) | 0.792 ± 0.009 (1224) | 0.906 ± 0.007 (920) | 0.561 ± 0.006 (304) | 0.945 ± 0.003 (458) |
| Tiller Number | 0.155 ± 0.005 (460) | 0.603 ± 0.014 (1224) | 0.617 ± 0.014 (921) | 0.515 ± 0.008 (304) | 0.779 ± 0.007 (457) |
| Panicle Length | 0.392 ± 0.012 (460) | 0.802 ± 0.009 (1224) | 0.910 ± 0.008 (919) | 0.571 ± 0.005 (304) | 0.937 ± 0.005 (459) |
| Proportion of Spikelets Filled | 0.039 ± 0.003 (460) | 0.470 ± 0.003 (1224) | 0.306 ± 0.005 (920) | 0.732 ± 0.003 (305) | 0.759 ± 0.003 (458) |
| Yield per Plant | 0.195 ± 0.010 (460) | 0.625 ± 0.007 (1224) | 0.672 ± 0.007 (919) | 0.587 ± 0.006 (304) | 0.745 ± 0.005 (459) |
| Potential Yield | 0.366 ± 0.014 (460) | 0.693 ± 0.011 (1224) | 0.811 ± 0.008 (920) | 0.545 ± 0.010 (304) | 0.824 ± 0.006 (458) |
| *Genomic GCA + SCA model* |  |  |  |  |  |
| Height | 0.403 ± 0.019 (460) | 0.798 ± 0.010 (1224) | 0.917 ± 0.007 (920) | 0.484 ± 0.014 (304) | 0.930 ± 0.005 (458) |
| Tiller Number | 0.174 ± 0.006 (460) | 0.589 ± 0.015 (1224) | 0.622 ± 0.015 (921) | 0.406 ± 0.011 (304) | 0.710 ± 0.012 (457) |
| Panicle Length | 0.427 ± 0.020 (460) | 0.790 ± 0.010 (1223) | 0.899 ± 0.010 (919) | 0.405 ± 0.017 (305) | 0.888 ± 0.010 (459) |
| Proportion of Spikelets Filled | 0.037 ± 0.003 (460) | 0.433 ± 0.003 (1224) | 0.310 ± 0.005 (920) | 0.668 ± 0.005 (304) | 0.688 ± 0.004 (458) |
| Yield per Plant | 0.247 ± 0.013 (460) | 0.624 ± 0.008 (1224) | 0.693 ± 0.008 (919) | 0.464 ± 0.012 (304) | 0.717 ± 0.006 (459) |
|  |  |  |  |  |  |
| **Table S4 (cont.)** |  |  |  |  |  |
| **Trait** | **T0** | **T1** | **T1F** | **T1M** | **T2** |
| Potential Yield | 0.391 ± 0.015 (460) | 0.682 ± 0.011 (1224) | 0.780 ± 0.010 (919) | 0.496 ± 0.013 (304) | 0.803 ± 0.007 (459) |

**Table S5** Genomic prediction model predictive ability and standard error for male GCA for each trait as estimated by ten-fold cross-validation.

| **Trait** | **Genomic GCA model** | **Genomic GCA + SCA model** |
| --- | --- | --- |
| Height | 0.225 ± 0.054 | 0.215 ± 0.062 |
| Tiller Number | 0.185 ± 0.051 | 0.217 ± 0.054 |
| Panicle Length | 0.199 ± 0.038 | 0.214 ± 0.036 |
| Proportion of Spikelets Filled | 0.313 ± 0.082 | 0.319 ± 0.076 |
| Yield per Plant | 0.166 ± 0.054 | 0.180 ± 0.059 |
| Potential Yield | 0.209 ± 0.056 | 0.164 ± 0.067 |

**Table S6.** Phenotypic reliabilities for male GCA.

| **Trait** | **GCA model** | **GCA + SCA model** |
| --- | --- | --- |
| Height | 0.655 | 0.393 |
| Tiller Number | 0.415 | 0.325 |
| Panicle Length | 0.463 | 0.064 |
| Proportion of Spikelets Filled | 0.742 | 0.637 |
| Yield per Plant | 0.410 | 0.316 |
| Potential Yield | 0.234 | 0.206 |

**Table S7.** Phenotypic reliabilities for female GCA.

| **Trait** | **GCA model** | **GCA + SCA model** |
| --- | --- | --- |
| Height | 0.820 | 0.820 |
| Tiller Number | 0.739 | 0.737 |
| Panicle Length | 0.829 | 0.828 |
| Proportion of Spikelets Filled | 0.806 | 0.767 |
| Yield per Plant | 0.728 | 0.722 |
| Potential Yield | 0.810 | 0.809 |

**Table S8** Top 20 predicted F_1_ hybrid genetic values of yield per plant (g) ranked by their genomic GCA and genomic GCA + SCA predicted values and the standard error of the prediction. The predicted genetic values of the F­_1_ from the GCA and GCA + SCA models and the standard errors of their prediction are also listed for lines in bold, which were phenotyped as well as genotyped.

| **Ranked by genomic GCA model estimate** | | | **Ranked by genomic GCA + SCA model estimate** | | |
| --- | --- | --- | --- | --- | --- |
| *Hybrid* | *Genomic Estimated  Genetic Value* | *Estimated Genetic Value* | *Hybrid* | *Genomic Estimated Genetic Value* | *Estimated Genetic Value* |
| **4A:wtr_386** | 40.771 ± 3.588 | 40.188 ± 4.358 | 4A:wtr_388 | 43.352 ± 5.435 |  |
| 4A:wtr_102 | 40.548 ± 3.490 |  | **4A:wtr_503** | 43.217 ± 6.256 | 42.035 ± 5.456 |
| 4A:wtr_373 | 40.048 ± 3.777 |  | **4A:wtr_386** | 43.186 ± 4.883 | 41.292 ± 5.498 |
| **4A:wtr_332** | 39.945 ± 3.123 | 41.904 ± 3.909 | **4A:wtr_181** | 42.238 ± 5.352 | 41.138 ± 5.336 |
| 4A:wtr_314 | 39.928 ± 3.235 |  | **4A:wtr_540** | 42.170 ± 4.974 | 41.765 ± 5.126 |
| **4A:wtr_248** | 39.850 ± 3.162 | 44.305 ± 4.223 | **4A:wtr_392** | 42.144 ± 5.387 | 40.117 ± 5.418 |
| 4A:wtr_469 | 39.796 ± 2.958 |  | **4A:wtr_165** | 42.087 ± 5.854 | 42.640 ± 5.735 |
| **4A:wtr_242** | 39.786 ± 2.923 | 41.338 ± 3.753 | 4A:wtr_206 | 41.921 ± 4.554 |  |
| 4A:wtr_399 | 39.743 ± 3.243 |  | 4A:wtr_373 | 41.739 ± 5.254 |  |
| 4A:wtr_385 | 39.638 ± 3.132 |  | **4A:wtr_562** | 41.593 ± 5.684 | 40.126 ± 5.735 |
| **4A:wtr_253** | 39.590 ± 3.028 | 42.306 ± 3.826 | **4A:wtr_336** | 41.559 ± 4.562 | 38.949 ± 5.316 |
| **4A:wtr_232** | 39.372 ± 3.187 | 40.365 ± 3.913 | **4A:wtr_60** | 41.358 ± 4.698 | 45.648 ± 5.256 |
| 4A:wtr_158 | 39.342 ± 2.950 |  | **4A:wtr_332** | 41.242 ± 4.212 | 42.633 ± 5.257 |
| 4A:wtr_206 | 39.260 ± 3.141 |  | 4A:wtr_277 | 41.181 ± 4.804 |  |
| **4A:wtr_395** | 39.217 ± 3.090 | 39.973 ± 3.827 | 4A:wtr_385 | 41.064 ± 4.315 |  |
| **4A:wtr_236** | 39.183 ± 2.870 | 40.827 ± 4.225 | 4A:wtr_102 | 41.016 ± 5.397 |  |
| **4A:wtr_212** | 39.181 ± 3.183 | 41.194 ± 3.825 | **4A:wtr_232** | 40.969 ± 4.337 | 41.227 ± 5.244 |
| 4A:wtr_211 | 39.171 ± 4.182 |  | **4A:wtr_492** | 40.865 ± 4.525 | 40.665 ± 5.633 |
| 4A:wtr_388 | 39.160 ± 3.937 |  | **4A:wtr_395** | 40.811 ± 4.236 | 41.050 ± 5.296 |
| **4A:wtr_336** | 39.143 ± 3.230 | 38.659 ± 3.908 | 4A:wtr_396 | 40.692 ± 5.841 |  |

**Table S9** Top 20 predicted F_1_ hybrid genetic values of potential yield per plant (g) ranked by their genomic GCA and genomic GCA + SCA predicted values and the standard error of the prediction. The predicted genetic values of the F­_1_ from the GCA and GCA + SCA models and the standard errors of their prediction are also listed for lines in bold, which were phenotyped as well as genotyped.

| **Ranked by genomic GCA model estimate** | | | | **Ranked by genomic GCA + SCA model estimate** | | |
| --- | --- | --- | --- | --- | --- | --- |
| *Hybrid* | *Genomic estimated genetic value* | *Estimated genetic value* | *Hybrid* | | *Genomic estimated genetic value* | *Estimated genetic value* |
| **4A:wtr_555** | 71.505 ± 4.694 | 68.812 ± 5.532 | **4A:wtr_540** | | 77.401 ± 6.647 | 72.373 ± 6.126 |
| **4A:wtr_562** | 70.582 ± 5.430 | 67.799 ± 5.675 | **4A:wtr_392** | | 77.271 ± 7.069 | 70.235 ± 6.254 |
| **4A:wtr_242** | 70.575 ± 3.794 | 71.146 ± 5.010 | **4A:wtr_562** | | 76.157 ± 7.439 | 68.893 ± 6.550 |
| 4A:wtr_211 | 70.172 ± 5.393 |  | 4A:wtr_388 | | 75.821 ± 6.972 |  |
| **4A:wtr_253** | 70.028 ± 3.902 | 71.524 ± 5.011 | **4A:wtr_555** | | 75.231 ± 6.189 | 70.200 ± 6.424 |
| **4A:wtr_387** | 70.026 ± 3.975 | 69.927 ± 5.095 | **4A:wtr_386** | | 73.015 ± 6.392 | 68.164 ± 6.424 |
| **4A:wtr_392** | 70.002 ± 4.969 | 68.712 ± 5.290 | 4A:wtr_563 | | 72.696 ± 7.088 |  |
| 4A:wtr_469 | 69.920 ± 3.838 |  | **4A:wtr_561** | | 72.448 ± 6.674 | 67.622 ± 6.371 |
| **4A:wtr_386** | 69.823 ± 4.831 | 67.352 ± 5.532 | **4A:wtr_307** | | 72.354 ± 5.894 | 70.257 ± 6.126 |
| 4A:wtr_290 | 69.798 ± 5.222 |  | **4A:wtr_529** | | 72.220 ± 6.665 | 68.784 ± 6.187 |
| **4A:wtr_492** | 69.771 ± 4.351 | 69.064 ± 5.404 | **4A:wtr_492** | | 71.804 ± 5.725 | 70.125 ± 6.334 |
| 4A:wtr_388 | 69.726 ± 5.136 |  | **4A:wtr_336** | | 71.660 ± 5.832 | 68.352 ± 6.064 |
| **4A:wtr_540** | 69.666 ± 4.643 | 69.369 ± 5.095 | **4A:wtr_387** | | 71.649 ± 5.253 | 70.790 ± 6.126 |
| **4A:wtr_289** | 69.539 ± 4.145 | 67.514 ± 5.532 | **4A:wtr_395** | | 71.415 ± 5.529 | 69.969 ± 6.225 |
| **4A:wtr_561** | 69.443 ± 4.836 | 67.967 ± 5.404 | **4A:wtr_181** | | 71.326 ± 7.057 | 68.510 ± 6.261 |
| **4A:wtr_173** | 69.314 ± 3.930 | 72.469 ± 5.094 | **4A:wtr_232** | | 71.283 ± 5.717 | 70.148 ± 6.261 |
| 4A:wtr_314 | 69.300 ± 4.245 |  | 4A:wtr_211 | | 71.170 ± 7.261 |  |
| **4A:wtr_332** | 69.276 ± 4.068 | 69.693 ± 5.095 | 4A:wtr_385 | | 71.138 ± 5.519 |  |
| 4A:wtr_399 | 69.212 ± 4.266 |  | 4A:wtr_290 | | 71.046 ± 6.865 |  |
| **4A:wtr_336** | 69.103 ± 4.141 | 68.254 ± 5.009 | **4A:wtr_503** | | 71.043 ± 8.186 | 67.657 ± 6.261 |

**Table S10** BLUPs of female GCAs (including the intercept) and their standard errors for yield per plant (g), ranked by their genomic GCA + SCA model predicted value. Lines in bold were phenotyped as well as genotyped.

| **Female** | **Genomic GCA Model** | **GCA Model** | **Genomic GCA + SCA Model** | **GCA + SCA model** |
| --- | --- | --- | --- | --- |
| **4A** | 36.522 ± 1.791 | 36.450 ± 2.195 | 36.341 ± 1.841 | 36.330 ± 2.211 |
| **A07** | 33.552 ± 1.853 | 33.150 ± 2.204 | 33.590 ± 1.868 | 33.138 ± 2.223 |
| 11A | 31.027 ± 2.996 |  | 30.969 ± 2.982 |  |
| 12A | 30.608 ± 4.323 |  | 30.539 ± 4.302 |  |
| 5A | 30.497 ± 3.719 |  | 30.446 ± 3.701 |  |
| 1A | 30.430 ± 2.564 |  | 30.386 ± 2.552 |  |
| 9A | 29.793 ± 4.133 |  | 29.745 ± 4.114 |  |
| 3A | 29.220 ± 3.624 |  | 29.176 ± 3.608 |  |
| 8A | 29.202 ± 4.046 |  | 29.159 ± 4.027 |  |
| **6A** | 28.952 ± 1.863 | 28.652 ± 2.290 | 28.945 ± 1.876 | 28.644 ± 2.313 |
| **7A** | 28.242 ± 1.914 | 28.305 ± 2.302 | 28.224 ± 1.934 | 28.326 ± 2.327 |
| **10A** | 27.673 ± 1.830 | 27.616 ± 2.213 | 27.619 ± 1.841 | 27.644 ± 2.231 |
| **2A** | 25.739 ± 1.846 | 25.626 ± 2.252 | 25.673 ± 1.857 | 25.571 ± 2.271 |

**Table S11** BLUPs of male GCAs (including the intercept) and their standard errors for yield per plant (g), ranked by their genomic GCA + SCA model predicted value. Lines in bold were phenotyped as well as genotyped.

| **Genomic GCA model** | | | **Genomic GCA + SCA model** | | |
| --- | --- | --- | --- | --- | --- |
| *Male* | *Genomic Estimated GCA* | *Estimated GCA* | *Male* | *Genomic Estimated GCA* | *Estimated GCA* |
| **wtr_386** | 34.252 ± 3.109 | 33.704 ± 3.766 | **wtr_102** | 34.047 ± 3.175 | 34.681 ± 3.193 |
| **wtr_102** | 34.029 ± 2.995 | 35.827 ± 3.235 | **wtr_248** | 33.431 ± 2.650 | 36.011 ± 3.417 |
| **wtr_373** | 33.529 ± 3.325 | 35.045 ± 4.400 | **wtr_332** | 33.414 ± 2.602 | 34.369 ± 3.183 |
| **wtr_332** | 33.426 ± 2.559 | 35.420 ± 3.234 | **wtr_242** | 33.269 ± 2.358 | 33.890 ± 3.070 |
| **wtr_314** | 33.409 ± 2.694 | 35.462 ± 3.613 | **wtr_469** | 33.095 ± 2.366 | 35.777 ± 3.333 |
| **wtr_248** | 33.331 ± 2.606 | 37.821 ± 3.607 | **wtr_158** | 33.061 ± 2.371 | 35.416 ± 3.090 |
| **wtr_469** | 33.277 ± 2.355 | 37.687 ± 3.470 | **wtr_314** | 33.059 ± 2.703 | 33.944 ± 3.449 |
| **wtr_242** | 33.267 ± 2.310 | 34.854 ± 3.045 | **wtr_373** | 33.048 ± 3.364 | 33.901 ± 3.949 |
| **wtr_399** | 33.224 ± 2.704 | 35.212 ± 3.779 | **wtr_386** | 32.977 ± 3.202 | 32.522 ± 3.609 |
| **wtr_385** | 33.119 ± 2.570 | 33.957 ± 3.356 | **wtr_253** | 32.905 ± 2.462 | 34.420 ± 3.095 |
| **wtr_253** | 33.072 ± 2.442 | 35.822 ± 3.135 | **wtr_206** | 32.865 ± 2.665 | 31.720 ± 3.040 |
| **wtr_232** | 32.854 ± 2.636 | 33.881 ± 3.239 | **wtr_232** | 32.830 ± 2.709 | 32.878 ± 3.245 |
| **wtr_158** | 32.823 ± 2.344 | 36.647 ± 3.135 | **wtr_236** | 32.814 ± 2.266 | 33.543 ± 3.389 |
| **wtr_206** | 32.741 ± 2.581 | 32.384 ± 3.044 | **wtr_399** | 32.741 ± 2.729 | 33.534 ± 3.616 |
| **wtr_395** | 32.698 ± 2.518 | 33.489 ± 3.135 | **wtr_336** | 32.719 ± 2.773 | 31.754 ± 3.158 |
| **wtr_236** | 32.664 ± 2.243 | 34.343 ± 3.610 | **wtr_385** | 32.709 ± 2.600 | 33.001 ± 3.278 |
| **wtr_212** | 32.662 ± 2.632 | 34.710 ± 3.133 | **wtr_250** | 32.495 ± 2.498 | 33.620 ± 3.205 |
| wtr_211 | 32.652 ± 3.779 |  | **wtr_470** | 32.479 ± 2.550 | 33.238 ± 3.552 |
| wtr_388 | 32.641 ± 3.506 |  | **wtr_377** | 32.421 ± 2.904 | 32.067 ± 3.805 |
| **wtr_336** | 32.624 ± 2.689 | 32.175 ± 3.233 | wtr_211 | 32.397 ± 3.817 |  |
